# Artifact-Controlled Multi-Vertebral Transfer Learning Ensemble for Patient-Level Three-Class Osteoporosis Screening Using Thoracoabdominal CT-Derived Lumbar Images

**DOI:** 10.3390/biomedicines14091988

**Published:** 2026-09-03

**Authors:** Bulent Yildiz, Emre Delibaş, Irfan Atik, Omer Abdulaziz Bilgec

**Affiliations:** 1 Department of Radiology, Faculty of Medicine, Sivas Cumhuriyet University, 58140 Sivas, Türkiye; irfanatik_91@hotmail.com (I.A.); omerbilgec3442@gmail.com (O.A.B.); 2 Department of Computer Engineering, Faculty of Engineering, Sivas Cumhuriyet University, 58140 Sivas, Türkiye; edelibas@cumhuriyet.edu.tr; 3 Artificial Intelligence Systems and Data Science Applications and Research Center, Sivas Cumhuriyet University, 58140 Sivas, Türkiye

**Keywords:** osteoporosis screening, lumbar CT, patient-level classification, multi-vertebral representation, artifact-controlled deep learning

## Abstract

**Background/Objectives**: Osteoporosis and osteopenia remain underdiagnosed despite routine CT examinations containing potentially useful bone information. This study evaluated the feasibility of patient-level three-class classification of Normal, Osteopenia, and Osteoporosis classes using lumbar vertebral PNG images derived from routine thoracoabdominal CT scans, without raw DICOM data, HU values, or quantitative BMD information. **Methods**: The dataset comprised 190 patients labeled by DXA-derived mean L1–L4 T-scores and 760 vertebral images. Four vertebral images per patient were analyzed jointly using patient-level 5-fold stratified cross-validation. ROI annotations defined only the standardized crop center and were not used as model inputs, masks, or features. ResNet18, ResNet50, and DenseNet121 transfer learning models were trained with patient-level L1–L4 feature fusion, and their probabilities were combined using equal-weight soft voting. **Results**: Within the patient-level 5-fold cross-validation framework (the same fold was used for model selection and final performance reporting, rather than serving as an independent held-out test set), the final ensemble reached a cross-validation accuracy of 0.7474 (95% CI: 0.6842–0.8053), balanced accuracy of 0.7377 (95% CI: 0.6703–0.8009), macro-F1 of 0.7403 (95% CI: 0.6730–0.8011), and macro-average ROC-AUC of 0.8576 (95% CI: 0.8091–0.9013). ROI occlusion reduced performance, whereas mask-only and geometry-only controls indicated that ROI annotation or geometry alone was insufficient for class discrimination. Grad-CAM showed that activations were not confined to ROI annotations, although residual ROI-related effects could not be fully excluded. **Conclusions**: Patient-level multi-vertebral transfer learning demonstrated moderate feasibility for image-based osteoporosis decision support, supporting a preclinical decision-support role rather than direct diagnosis or replacement of QCT/BMD assessment.

## 1. Introduction

Osteoporosis is a systemic skeletal disease characterized by reduced bone mineral density and deterioration of bone microarchitecture. These changes weaken trabecular and cortical bone structure and increase susceptibility to fragility fractures. Vertebral, hip, and distal radius fractures are associated with morbidity, mortality, loss of independence, reduced quality of life, and increased healthcare costs. With population aging, osteoporosis and osteopenia have become increasingly important not only for individual patient care, but also for public health and healthcare resource sustainability [1,2].

Dual-energy X-ray absorptiometry (DXA) is the widely accepted primary reference method for assessing bone mineral density. According to the World Health Organization (WHO), osteoporosis is defined as a T-score of −2.5 or lower, whereas osteopenia is defined as a T-score between −1.0 and −2.5 [3,4]. Despite the clinical value of DXA, osteoporosis screening and treatment rates remain suboptimal in many healthcare systems. Limited access to DXA, referral barriers, additional appointment requirements, low clinical awareness, and the often asymptomatic course of the disease before fracture contribute to delayed diagnosis [1,2]. These limitations have increased interest in opportunistic screening approaches that extract additional information from imaging examinations already obtained during routine clinical care.

Computed tomography (CT) is widely used for diverse clinical indications and may provide additional information about bone status without requiring new imaging or additional radiation exposure. Opportunistic CT-based osteoporosis assessment has been developed around vertebral Hounsfield Unit (HU) measurements, Quantitative Computed Tomography (QCT)-based volumetric bone mineral density analysis, CT-derived image biomarkers, and machine learning-based classification models [5,6]. HU values measured from lumbar CT regions of interest have been reported to correlate with DXA and to help differentiate osteoporosis or osteopenia [7]. Machine learning models combining HU measurements with demographic variables have also been proposed as triage tools, particularly for preoperative spine surgery planning and the identification of patients who may require further evaluation [8]. However, HU- and QCT-based approaches depend on raw DICOM data, calibration, acquisition parameters, ROI standardization, and measurement reliability. Their direct use may therefore be limited when only exported images or non-DICOM formats without quantitative density information are available.

Artificial intelligence and deep learning have become active research areas in opportunistic osteoporosis screening. Deep learning models have been applied to vertebral localization, segmentation, bone mineral density estimation, normal bone density–osteopenia–osteoporosis classification, and fracture-risk stratification. Systematic reviews and meta-analyses suggest that deep learning-based osteoporosis prediction can achieve promising diagnostic performance, while also emphasizing heterogeneity across studies, limited generalizability, and the need for external validation [9]. Recent scoping reviews and CT image-based classification and prediction studies similarly show that AI-assisted opportunistic osteoporosis screening is expanding across different imaging settings and clinical workflows [10,11].

Much of the CT-based deep learning literature relies on DICOM- or QCT-derived quantitative density information. Oh et al. developed automated DL-QCT/DL-BMD measurements at the L1–L2 vertebral levels in routine CT scans and reported strong agreement with manual QCT BMD values [12]. Park et al. evaluated automated DL-BMD in a multicenter dataset with different CT protocols and scanner manufacturers, reporting high correlation with manual BMD and high diagnostic performance against a QCT-based reference [13]. These studies support the potential of AI-assisted CT analysis for automated and scalable osteoporosis screening. However, they depend on preservation of DICOM/QCT-based density information and the ability to produce BMD-like quantitative measurements, which differs from classification using non-DICOM image exports.

CT-based deep learning has also been used for direct classification. Pan and colleagues developed a DCNN-based opportunistic screening model for distinguishing normal, osteopenia, and osteoporosis classes using L1 and L2 vertebral information from chest CT images [14]. In a related multi-feature fusion study, the same group combined L1 vertebral images, clinical variables, and radiomic features for three-class bone density classification and volumetric BMD estimation [15]. Zhang et al. proposed an end-to-end multitask joint learning model that combined localization, segmentation, and classification for three-class osteoporosis assessment [16]. These studies show that the three-class problem is clinically meaningful, but often requires more complex modeling pipelines involving segmentation, localization, density estimation, or multimodal data.

Transfer learning remains relevant in smaller medical imaging datasets. Dzierżak and Omiotek compared pre-trained CNN architectures on CT images of L1 trabecular bone tissue and showed that transfer learning can be applied to osteoporosis classification under limited data conditions [17]. Radiomics-based machine learning studies have also explored lumbar CT for osteoporosis screening. Gao et al. reported that an XGBoost model using selected PyRadiomics features outperformed HU- and VBQ-based models [18]. Together, these findings suggest that osteoporosis-related information may be reflected not only in average density measurements, but also in heterogeneity, texture, and vertebral morphological patterns.

Osteoporosis-related AI research is not limited to CT. Transformer-based multiscale fusion models on lumbar spine X-ray images have shown that combining texture and local features may help distinguish normal bone density, osteopenia, and osteoporosis [19]. Transfer learning with image enhancement and fuzzy fusion has also been applied to X-ray-based osteoporosis prediction [20]. In addition, multimodal deep learning models combining chest X-ray images with clinical data have shown that age, sex, biochemical markers, and other clinical variables can provide complementary information for osteoporosis prediction [21]. These studies demonstrate the value of multimodal information; however, when data sharing is limited to anonymized images and clinical variables are unavailable, pure image-based approaches still need to be evaluated separately.

DXA itself may also be affected by specific clinical conditions. Osteophytes, endplate sclerosis, abdominal aortic calcification, and degenerative changes can lead to overestimation of lumbar DXA BMD. In such settings, CT-based trabecular assessment may more directly reflect bone status. Wu et al. reported that deep learning-based trabecular BMD estimation from lumbar CT improved fracture-risk discrimination compared with DXA in patients with hyperostosis or ligamentous calcification [22]. However, this level of clinical inference requires quantitative BMD estimation and appropriate reference validation. Studies based only on non-DICOM PNG images cannot make quantitative claims at this level.

Overall, current AI-based osteoporosis research has mainly advanced along three lines: HU/QCT-based quantitative densitometry and BMD regression, automated opportunistic screening with vertebral localization or segmentation, and transfer learning or feature-fusion approaches for direct classification from X-ray, CT, or multimodal data. These studies show that routine clinical images can contain useful information about bone health. Nevertheless, several practical gaps remain. Many studies rely on raw DICOM data, HU, QCT, or BMD information; some focus on binary classification; and others use a single vertebral level or image-level evaluation. Evidence remains limited for three-class, non-DICOM PNG-based classification using L1–L4 images jointly at the patient level while explicitly controlling for annotation-related artifacts.

The present study addresses this narrower but practically relevant data scenario. It does not aim to replace QCT, HU measurement, DL-BMD estimation, or DXA. Instead, it investigates whether Normal, Osteopenia, and Osteoporosis categories can be distinguished at the patient level from PNG-format lumbar vertebral images derived from routine thoracoabdominal CT scans, without raw DICOM data, true HU values, or quantitative BMD information. In this sense, the study is positioned as a feasibility analysis of image-based decision support using patient-level multi-vertebral representation in a non-DICOM image format. Accordingly, the study is not intended to compete with simple HU measurement when DICOM data are available; rather, it evaluates whether a patient-level image-based signal can still be extracted when the available data are limited to non-DICOM CT-derived PNG images.

Each patient was represented by four images corresponding to the L1, L2, L3, and L4 vertebral levels. Each vertebral image was processed using a shared CNN backbone, the resulting vertebral-level features were fused at the patient level, and a single patient-level class prediction was produced. Throughout training and evaluation, all L1–L4 images from the same patient were kept within the same fold to prevent patient-level data leakage that could arise from image-level random partitioning. This design is particularly important in medical imaging studies with limited patient numbers, where leakage can easily lead to overestimated performance.

In this study, ResNet18-, ResNet50-, and DenseNet121-based transfer learning models were developed with patient-level L1–L4 feature fusion. Patient-level probabilities from these models were then combined using an equal-weight soft-voting ensemble. Because different CNN backbones may respond differently to vertebral image patterns, ensemble modeling was used to assess whether a more balanced patient-level prediction could be obtained than with a single backbone. The evaluation unit was the patient rather than the individual image; therefore, model outputs were interpreted as patient-level predictions based on 190 patients, not as image-level predictions based on 760 images.

A further methodological issue was the presence of visible ROI annotations on the PNG images. These annotations were created during routine clinical assessment and were not provided to the model as masks, additional channels, or classification features. They were used only as anatomical references to determine the standardized crop center. Because removing the ROI line by inpainting could introduce synthetic texture, the annotations were retained, but their possible shortcut-learning effect was explicitly evaluated. ROI occlusion, mask-only and geometry-only negative controls, and Grad-CAM-based artifact-control analyses were therefore included to examine whether model performance could be explained solely by ROI annotation or ROI geometry.

The aim of this study was to evaluate the patient-level discriminability of Normal, Osteopenia, and Osteoporosis classes using PNG-format L1–L4 lumbar vertebral images derived from routine thoracoabdominal CT scans. Patient labels were based on DXA-derived mean L1–L4 T-score values and used as clinical reference classes. The study was not designed as a BMD estimator, HU measurement method, or QCT alternative. Rather, it was designed as an artifact-controlled feasibility study of three-class osteoporosis screening and decision support using patient-level multi-vertebral transfer learning on CT-derived images lacking DICOM density information.

## 2. Material and Methods

### 2.1. Study Cohort, Patient Selection, and Reference Standard

This study was designed as a retrospective, observational, non-interventional medical image analysis study. The image data consisted of lumbar vertebral CT-derived images that had been obtained previously as part of routine clinical care; no additional imaging, invasive procedure, drug administration, or clinical follow-up was performed for the purposes of this study. All images and class labels were transferred to the research team after anonymization. The study was approved by the Sivas Cumhuriyet University Health Sciences Research Ethics Committee (decision no: 2026-07/60; date: 16 July 2026).

Patient selection was performed retrospectively through the hospital Picture Archiving and Communication System (PACS). Initially, 1754 adult patients who had undergone DXA for any clinical indication were screened. Among these patients, 229 patients who also had an available thoracoabdominal CT examination within six months of the DXA examination were identified and considered eligible for further evaluation.

**Figure 1:**
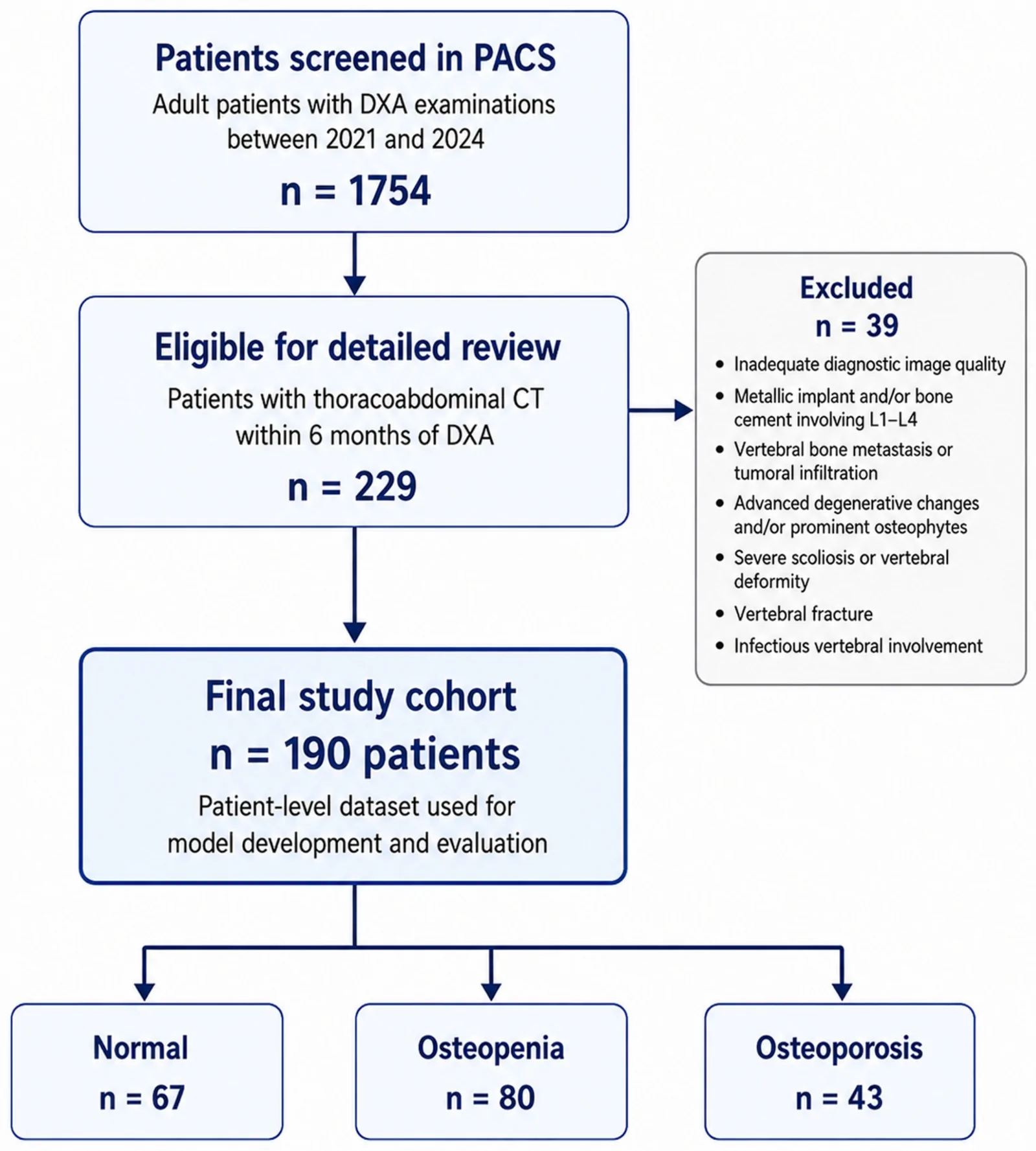
Patient selection flowchart. The flowchart summarizes the screening, eligibility assessment, exclusion process, and final diagnostic group distribution of the study cohort.

The inclusion criteria were as follows: (1) availability of both DXA and thoracoabdominal CT examinations within a six-month interval; (2) availability of CT images adequately covering the L1–L4 vertebral levels; and (3) sufficient CT image quality for vertebral assessment and image preparation. Patients were excluded if they had inadequate diagnostic image quality (*n* = 5), metallic implant and/or bone cement involving the L1–L4 vertebral levels (*n* = 4), vertebral bone metastasis or tumoral infiltration (*n* = 6), advanced degenerative changes and/or prominent osteophytes (*n* = 12), severe scoliosis or vertebral deformity (*n* = 4), vertebral fracture (*n* = 5), or infectious involvement of the vertebrae (*n* = 3). A total of 39 patients were excluded based on these predefined criteria, and the final study cohort consisted of 190 patients. The overall patient selection, exclusion, and final cohort formation process is summarized in Figure 1.

The study cohort consisted of adult patients who underwent thoracoabdominal CT examinations for routine clinical indications between 2021 and 2024 and who also had bone mineral density assessment by DXA during the same period. Therefore, the images used in this study were not obtained from dedicated lumbar spine CT examinations. Instead, they were derived from the L1–L4 lumbar vertebral levels included within the scan range of routine thoracoabdominal CT examinations. This design supports the interpretation of the study in the context of opportunistic osteoporosis screening. The time interval between CT and DXA examinations was limited to a maximum of six months, and patients with longer intervals were excluded. In the final cohort, the mean CT–DXA interval was 42.6 ± 28.4 days, with a range of 0–180 days. The corresponding mean intervals were 39.8 ± 26.1 days in the Normal group, 44.2 ± 29.7 days in the Osteopenia group, and 43.9 ± 29.1 days in the Osteoporosis group, with no statistically significant difference among the groups (*p* = 0.412).

The study used a three-class image dataset constructed from L1–L4 lumbar vertebral PNG images derived from thoracoabdominal CT examinations. Patient-level class labels were determined according to the standard L1–L4 composite/mean T-score automatically reported by the Hologic DXA system, rather than by visual or subjective evaluation of the CT images. The L1–L4 T-score was not manually calculated by the investigators as a separate arithmetic mean of individual vertebral T-scores. This lumbar spine composite/mean T-score was selected because the image-based model also used the L1–L4 vertebral levels jointly at the patient level, allowing the imaging representation and the DXA-based reference label to correspond anatomically.

Patients were categorized according to World Health Organization criteria into the Normal, Osteopenia, or Osteoporosis class. Patients with an L1–L4 T-score above −1.0 were classified as Normal, those with a T-score between −1.0 and −2.5 as Osteopenia, and those with a T-score of −2.5 or lower as Osteoporosis. These categories were used as patient-level clinical reference labels during model development and evaluation.

Each patient in the dataset was represented by four images corresponding to the L1, L2, L3, and L4 vertebral levels. This structure allowed complementary anatomical and textural information from multiple lumbar levels to be used together, rather than relying on a single vertebral level. Accordingly, the dataset was organized at the patient level rather than at the image level, and all L1–L4 images belonging to the same patient were handled as a single patient record throughout the analyses. The final dataset consisted of 190 patients and 760 images. Of these patients, 67 were classified as Normal, 80 as Osteopenia, and 43 as Osteoporosis. Patient characteristics, class distribution, sex distribution, and CT–DXA time intervals are presented in Table 1. The anonymized study dataset did not include any information that could directly or indirectly identify patients, such as names, protocol numbers, identification numbers, or dates. The modeling dataset consisted only of anonymized patient codes, patient-level class labels, and L1–L4 vertebral image files.

The CT–DXA interval ranged from 0 to 180 days in the overall cohort. There was no statistically significant difference in the CT–DXA interval among the three diagnostic groups (*p* = 0.412). Age and sex were reported for cohort description and were not used as model inputs. The age distributions were similar across the three diagnostic groups. The cohort was predominantly female in all groups, consistent with the clinical epidemiology of osteopenia and osteoporosis. Demographic variables were reported to describe the study cohort; however, age and sex were not included as input variables in the present image-based model.

**Figure 2:**
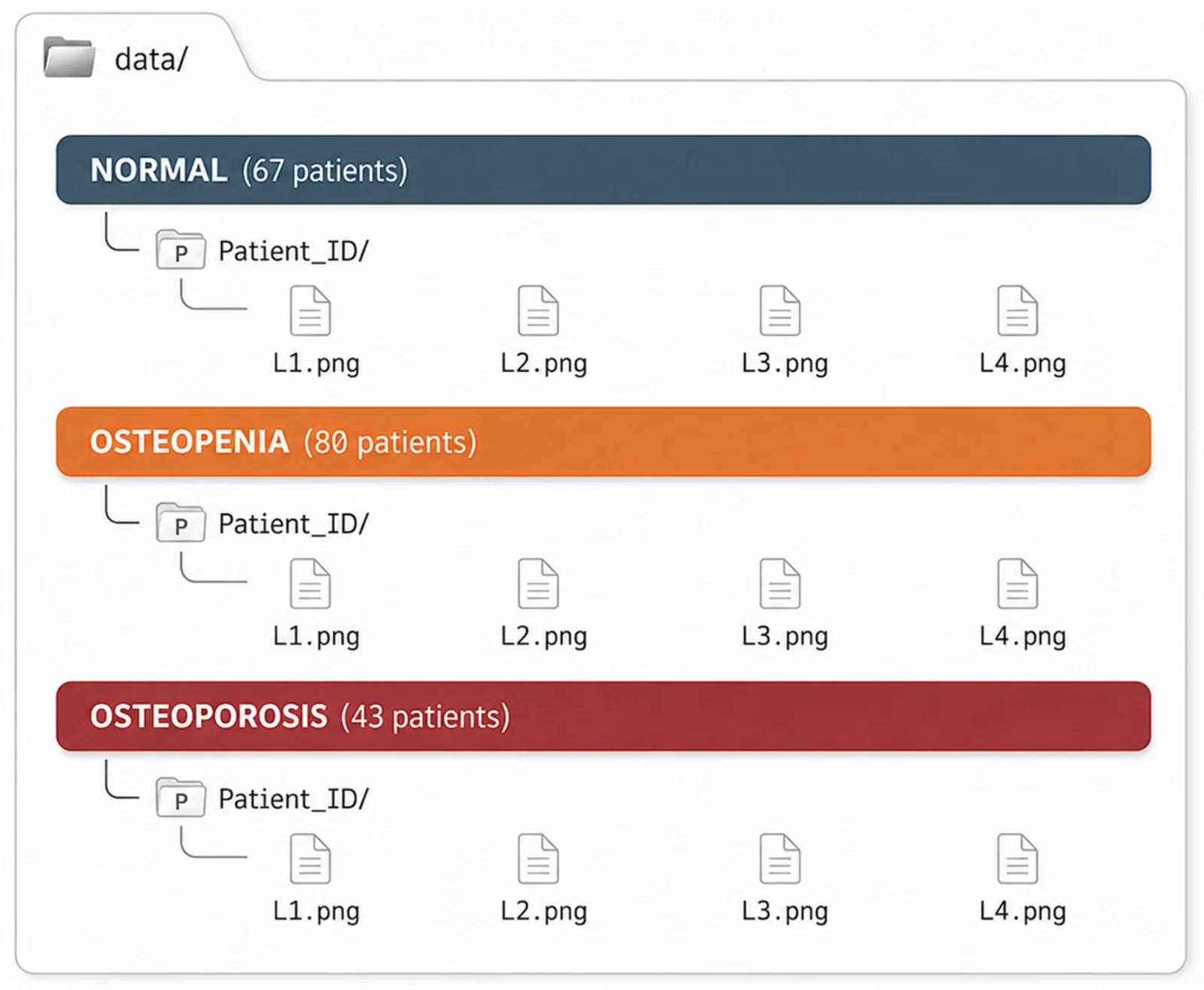
Patient-level dataset organization. The dataset was organized hierarchically by class, patient, and vertebral level. L1–L4 images from the same patient were kept together in all analyses.

The dataset folder structure was arranged to support this patient-level organization. Each class was stored in a separate main folder, each patient was represented by an individual subfolder, and the corresponding L1–L4 images were saved as separate files within that patient folder. This structure enabled patient-level cross-validation in the subsequent analyses and ensured that images from the same patient were not split between the training and validation sets. The dataset organization is shown in Figure 2.

### 2.2. Image Characteristics

The images used in this study were not raw DICOM slices, but lumbar vertebral CT-derived images exported in PNG format during clinical assessment. Each image contained a circular region of interest (ROI) annotation placed for trabecular bone assessment at the corresponding vertebral level. This ROI annotation was not used as a classification target, segmentation mask, additional model input, or explicit feature. It was used only as an anatomical reference for standardizing vertebral localization.

The source CT examinations consisted of thoracoabdominal CT scans acquired at the same institution between 2021 and 2024 using a consistent imaging protocol. All CT examinations were performed on a single 128-detector multidetector CT scanner (Revolution EVO; GE Healthcare, Milwaukee, WI, USA). The protocol used a tube voltage of 120 kVp, automatic tube current modulation (Smart mA, GE Healthcare), a gantry rotation time of 0.5 s, and reconstruction with a slice thickness of 0.625 mm. Therefore, scanner- and protocol-related variability was intentionally limited in the present dataset, but robustness to other scanners, reconstruction settings, or acquisition protocols could not be assessed. For the modeling dataset, anonymized axial L1–L4 vertebral images were selected during clinical assessment and exported in PNG format.

The use of PNG images defines an important technical boundary of this study. Although the source CT examinations were acquired using a single scanner and standardized protocol, the exported PNG images did not preserve true DICOM-based Hounsfield unit (HU) values, windowing parameters, or patient- and image-level quantitative metadata. Therefore, HU-based quantitative measurement, QCT-like BMD estimation, and BMD regression were not performed. The proposed approach was accordingly evaluated as an image-based three-class screening and decision-support feasibility analysis using CT-derived PNG images.

Because of this data format, the model could use only visual information such as grayscale intensity distribution, trabecular bone appearance, cortical boundaries, and vertebral morphology. The preprocessing strategy therefore preserved the vertebral body together with its trabecular and cortical context rather than isolating only the narrow ROI interior. Since the ROI annotation remained visible, its possible artifact effect was examined through additional sensitivity, negative control, and visual explainability analyses.

**Figure 3:**
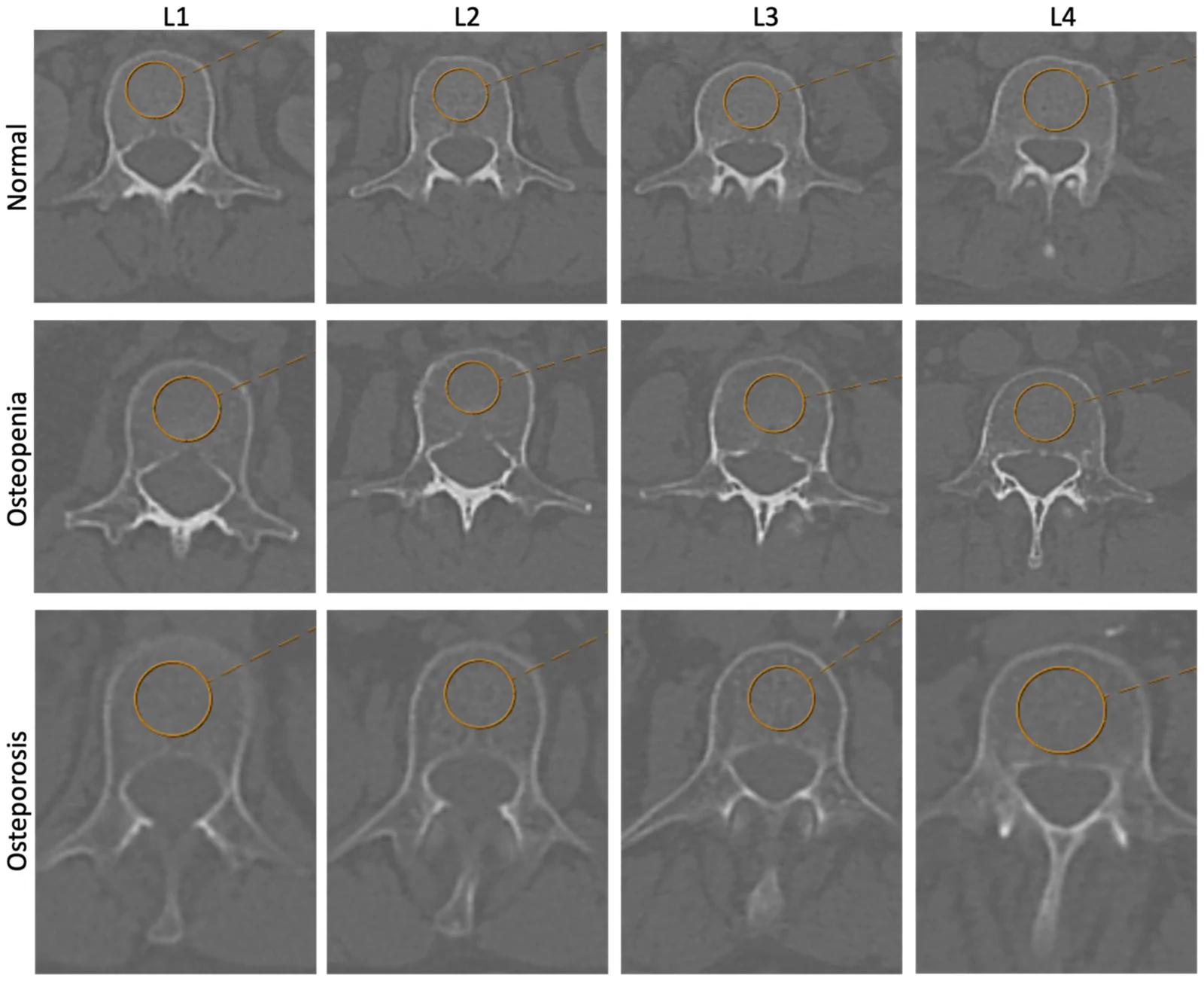
Representative L1–L4 lumbar vertebral images from example patients in each class. The images consist of CT-derived PNG slices exported during clinical assessment and include visible ROI annotations. The orange circular/elliptical line indicates the manually placed region-of-interest annotation used during clinical trabecular bone assessment; it was not used as a classification target, segmentation mask, additional model input, or explicit feature in the proposed model.

Figure 3 shows representative L1–L4 images from one example patient in each class. These images illustrate the PNG format of the dataset, the visible ROI annotations, and the anatomical variability across vertebral levels.

### 2.3. Dataset Integrity and Validation

Before model development, automated integrity and consistency checks were performed on the dataset. These checks included verification of the class folders, patient subfolders, the presence of L1–L4 image files for each patient, and the readability of all image files. The inspection confirmed that all patients had complete images for the four vertebral levels and that there were no corrupted or unreadable image files.

Because ROI-guided cropping was planned, the detectability of ROI annotations on the images was also assessed before preprocessing. ROI annotations were searched in each image using HSV color-space-based masking followed by connected-component analysis, and an ROI annotation was detected in all images. This step was not used to generate classification features; rather, it was used to localize the vertebral body according to a standardized anatomical reference point in the subsequent cropping stage.

To support patient-level evaluation and prevent image-level leakage, the dataset was divided into five stratified folds at the patient level. All L1–L4 images from the same patient were assigned to the same fold, and the same fold structure was used for all model training, ensemble, and performance analyses. Class distribution was preserved during fold assignment, and each fold included 38 patients, as shown in Table 2.

### 2.4. ROI-Guided Standardized Vertebral Cropping

The original PNG images varied across patients and vertebral levels in terms of position, field of view, and surrounding anatomical content. Therefore, standardized vertebral crops were generated before model development. The aim of cropping was not to isolate only the ROI interior, but to represent the vertebral body together with trabecular structure, cortical boundaries, and adjacent anatomical context.

The circular ROI annotation on each image was used as an anatomical reference for determining the crop center. The ROI was not used as a separate mask, label, or additional feature for classification. ROI detection was performed using color-based masking in the HSV color space, followed by connected-component analysis to estimate the center of the annotated region. During visual quality checks, the ROI center was observed to lie slightly superior to the vertebral trabecular center in some images. Therefore, the crop center was shifted 20 pixels inferiorly relative to the detected ROI center. This shift was defined as a fixed anatomical preprocessing parameter and was not optimized according to model performance.

A fixed 320 × 320-pixel crop was extracted from each image. When the crop window approached the image borders, the window was translated so that it remained within the original image boundaries. No zero padding, reflection padding, edge replication, or other artificial pixel-generation method was applied. Thus, all model inputs were composed only of original image content. This ROI-guided cropping strategy was applied consistently across all classes, patients, and vertebral levels.

**Figure 4:**
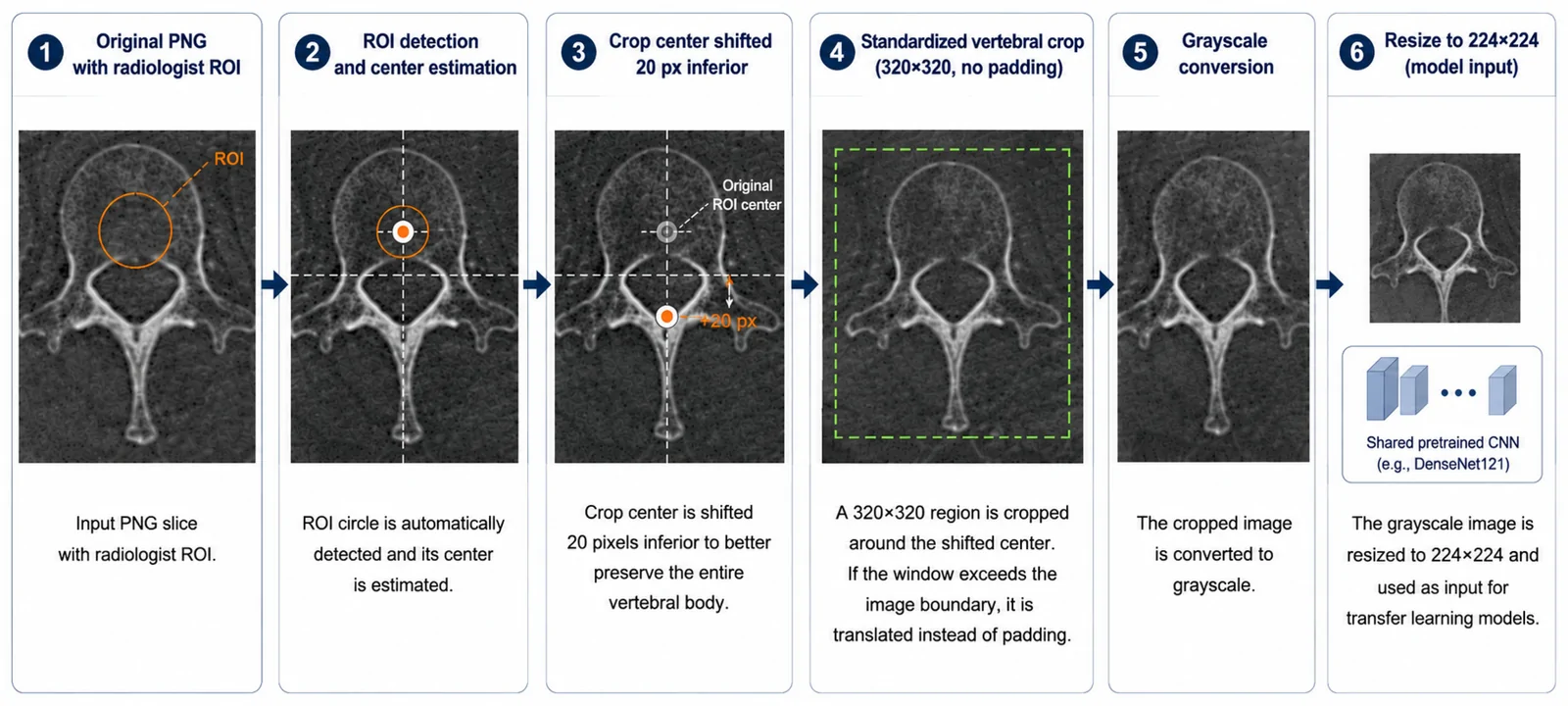
ROI-guided standardized vertebral cropping workflow. The ROI annotation was detected using HSV-based masking, the crop center was shifted 20 pixels inferiorly, and a 320 × 320 vertebral crop was obtained.

**Figure 5:**
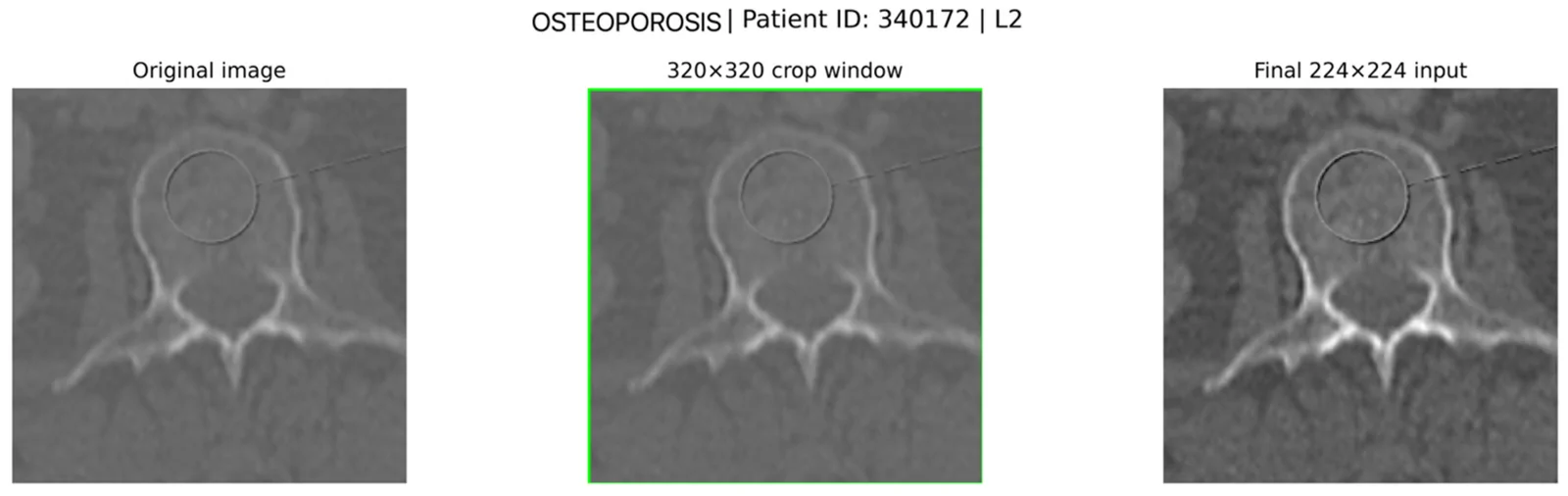
Example preprocessing steps from the original image to the model input. ROI center detection, 20-pixel inferior crop-center shifting, 320 × 320 vertebral cropping, and standardized model input generation are shown. No artificial padding was applied during cropping.

At the end of preprocessing, four standardized vertebral crops corresponding to L1, L2, L3, and L4 were obtained for each patient and used together for patient-level multi-vertebral modeling. The ROI-guided cropping workflow is shown in Figure 4, and an example preprocessing result is shown in Figure 5.

### 2.5. Rationale for Preserving ROI Annotations

The PNG images used in this study contained circular ROI annotations placed at the L1, L2, L3, and L4 vertebral levels by a single experienced radiologist during radiological assessment. The ROIs were positioned on axial slices to represent the trabecular region of the vertebral body while avoiding the cortical bone and vascular plexus. These annotations were not used as separate masks, additional channels, or classification features. They served only as anatomical references for determining the standardized crop center.

After cropping, the ROI annotations were retained on the images. Removing the ROI line by inpainting or similar methods would not restore the original trabecular tissue and could introduce synthetic texture into the model input. Preserving the ROI line was therefore considered a more transparent preprocessing choice than replacing it with artificially reconstructed image content. At the same time, retaining ROI annotations may introduce a visual artifact and potential shortcut-learning risk. For this reason, their possible influence on model decisions was evaluated through ROI occlusion, mask-only and geometry-only negative controls, and Grad-CAM-based attention analysis, as described in Section 2.12.

### 2.6. Image Standardization

After ROI-guided cropping, the resulting 320 × 320-pixel vertebral images were standardized for model input. The images were first converted to grayscale and then resized to 224 × 224 pixels. This size was selected as the standard input dimension compatible with the ImageNet-pretrained CNN architectures used in the study. No additional image enhancement procedures, such as histogram equalization, CLAHE, sharpening, denoising, or contrast enhancement, were applied during preprocessing. This choice was made to standardize image size and channel structure while avoiding artificial modification of intensity and texture patterns.

Although the images were prepared in grayscale, they were converted to a three-channel format at the model stage to match the input structure of CNN models pretrained on ImageNet. This conversion did not generate new image information; it only represented the same grayscale content across three channels. Thus, pretrained ResNet and DenseNet backbones could be used without architectural modification. The same standardization procedure was applied consistently across all classes, patients, and vertebral levels. The final model input for each patient consisted of four standardized 224 × 224-pixel images corresponding to the L1, L2, L3, and L4 vertebral levels.

### 2.7. CNN Backbones and Patient-Level Feature-Fusion Models

Each patient was represented by four standardized images corresponding to the L1, L2, L3, and L4 vertebral levels. These four images were processed together to generate a single patient-level class prediction.

Three ImageNet-pretrained CNN backbones were evaluated for patient-level classification: ResNet18, ResNet50, and DenseNet121. These architectures were selected because they differ in network depth and connectivity structure, allowing the same L1–L4 patient-level representation to be assessed under different CNN backbones. In each model, L1, L2, L3, and L4 images were processed through the same shared CNN backbone, and the resulting vertebra-level feature representations were fused at the patient level. The fused representation was then passed through fully connected classifier layers to produce probability outputs for the Normal, Osteopenia, and Osteoporosis classes.

In the ResNet18 model, a 512-dimensional feature vector was obtained from each vertebral image, resulting in a 2048-dimensional concatenated patient-level feature vector. In the ResNet50 model, the same procedure was applied using 2048-dimensional vertebra-level features, yielding an 8192-dimensional patient-level representation. In the DenseNet121 model, each vertebral level was represented by a 1024-dimensional feature vector, producing a 4096-dimensional patient-level representation. In all models, the concatenated patient-level feature vector was fed into a classifier consisting of two intermediate fully connected layers.

**Figure 6:**
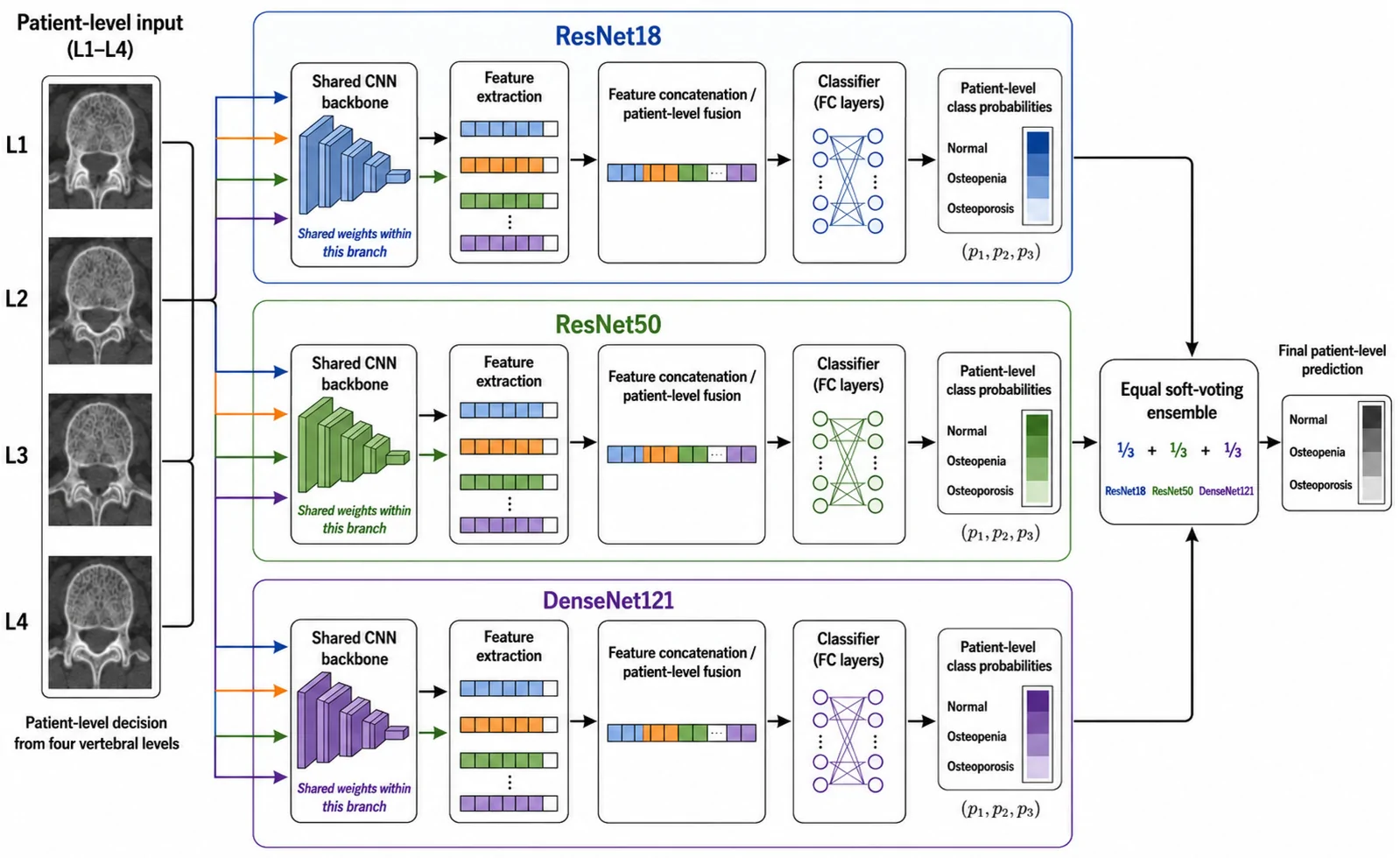
Patient-level L1–L4 feature-fusion and ensemble architecture. L1–L4 images from each patient were processed with a shared CNN backbone, concatenated at the patient level, and converted into class probabilities. ResNet18, ResNet50, and DenseNet121 probabilities were combined using equal-weight soft voting.

The patient-level L1–L4 feature-fusion and ensemble architecture is illustrated in Figure 6, and the feature-fusion structures of the CNN backbones are summarized in Table 3.

### 2.8. Training Protocol and Hyperparameters

All models were trained using the predefined patient-level 5-fold stratified cross-validation structure described in Section 2.3. In each fold, the training and validation/testing partitions were defined at the patient level.

To address class imbalance, weighted cross-entropy loss was used during training. Class weights were calculated from the training portion of each fold only, without using samples from the validation fold. The AdamW algorithm was used for optimization. Learning rate, weight decay, dropout rate, maximum number of epochs, and early stopping patience were defined according to the model architecture.

Data augmentation was deliberately limited. During training, small-angle rotations and mild brightness/contrast variations were applied. Horizontal and vertical flipping were not used in order to preserve clinical export orientation and avoid artificial changes in the spatial relationship between the visible ROI annotation and the crop reference. No data augmentation was applied during validation. In each fold, the best training epoch/checkpoint for each CNN model was selected according to validation macro-F1, because class imbalance made accuracy alone insufficient for model selection. The same validation fold was subsequently used for final fold-level performance reporting. Therefore, although no patient or image was shared between the training and validation partitions, best-epoch selection and final performance evaluation were not statistically independent, and the resulting fold-level metrics should not be interpreted as estimates from a strictly independent held-out test set. The implications of this design for performance interpretation are discussed in Section 4.5.

The training hyperparameters used for the CNN models are summarized in Table 4.

### 2.9. Computing Environment and Implementation Details

All data preprocessing, model training, inference, ensemble analysis, artifact-control experiments, and visual explainability analyses were performed in a Python-based computing environment (version 3.12.3). PyTorch version 2.12.1+cu130 and torchvision version 0.27.1+cu130 were used to develop the deep learning models. NumPy, pandas, and scikit-learn were used for data handling, table generation, and performance analysis. Image preprocessing, ROI masking, and image transformation operations were implemented using OpenCV- and PIL-based functions. Performance outputs, confusion matrices, and Grad-CAM visualizations were generated using Matplotlib version 3.11.0.

The experiments were conducted on a workstation running Ubuntu 24.04. The system was equipped with an Intel Core Ultra 7 processor (Intel Corporation, Santa Clara, CA, USA) and two NVIDIA RTX 5000 Pro Blackwell GPUs (NVIDIA Corporation, Santa Clara, CA, USA), each with 48 GB of memory. Model training and inference were performed using CUDA-enabled GPU acceleration.

To improve experimental reproducibility, a fixed random seed value of 42 was used in data splitting and modeling steps involving randomness. The complete experimental workflow, including preprocessing, fold assignment, training, inference, ensemble construction, statistical analysis, and visualization, was implemented using predefined scripts and the same patient-level fold identifiers across all analyses.

### 2.10. Ensemble Strategy and Meta-Learner Controls

The individual CNN models produced patient-level three-class probability vectors for the Normal, Osteopenia, and Osteoporosis classes. As the primary ensemble approach, the patient-level probability outputs of ResNet18, ResNet50, and DenseNet121 were averaged using equal weights. The final ensemble probability vector was calculated as follows:

(1) $$p_{ensemble}=\frac{1}{3}p_{ResNet18}+\frac{1}{3}p_{ResNet50}+\frac{1}{3}p_{DenseNet121}$$

where $p_{ResNet18}$, $p_{ResNet50}$, and $p_{DenseNet121}$ denote the three-class probability vectors produced by the corresponding models for the same patient. The final class prediction was obtained by selecting the class with the highest probability in the ensemble probability vector.

In addition to equal-weight soft voting, alternative combination strategies were evaluated to test whether more complex model fusion provided additional benefit. These included limited manually defined weighting schemes, temperature calibration, class-based weighting, and leakage-free logistic regression stacking. In the stacking experiments, the meta-learner was trained only on out-of-fold probabilities from the other four folds and then applied to the held-out fold. This design was used to avoid data leakage at the meta-learner level.

The final ensemble strategy was selected by considering patient-level performance, simplicity, transparency, and the use of patient-level partitions without inter-partition patient overlap. As reported in Section 3, the more complex combination strategies did not outperform the equal-weight soft-voting ensemble.

### 2.11. Performance Evaluation Metrics

Unless otherwise specified, all performance metrics reported in this study—including accuracy, balanced accuracy, macro-F1, and ROC-AUC—represent patient-level 5-fold cross-validation performance estimates. In the implemented design, the held-out fold was used both for best-epoch/checkpoint selection and for fold-level performance reporting; therefore, these estimates should not be interpreted as performance obtained from a statistically independent internal test set. Model performance was evaluated at the patient level. For each patient, the predicted class was compared with the corresponding clinical reference class. Accuracy, balanced accuracy, macro-precision, macro-recall, macro-F1, weighted-F1, and class-wise precision, recall, and F1 scores were calculated. Because of class imbalance, model comparisons were not based on accuracy alone; macro-F1 and class-wise recall were considered important for interpretation, particularly for the smaller Osteoporosis class.

For the final equal-weight soft-voting ensemble, probability-level discriminative performance was evaluated using one-vs-rest receiver operating characteristic (ROC) analysis. Class-wise AUC values were calculated for the Normal, Osteopenia, and Osteoporosis classes, together with macro-average and weighted-average ROC-AUC values.

To quantify sample-related uncertainty, patient-level bootstrap analysis was performed. The sampling unit was the patient rather than the individual image. Bootstrap 95% confidence intervals were calculated for the main performance metrics and ROC-AUC values. For paired model comparisons, exact McNemar tests were used for accuracy differences, and paired patient-level bootstrap confidence intervals were used to interpret differences in macro-F1 and other threshold-dependent metrics.

A confusion matrix was reported to summarize the distribution of errors across classes. Artifact-control analyses and Grad-CAM-based visual explainability assessments were also performed on the final model to evaluate the possible influence of ROI annotations.

### 2.12. Artifact-Control and Visual Explainability Protocol

Because ROI annotations were retained on the images, additional artifact-control analyses were performed on the final ensemble model to evaluate whether model performance could be explained by annotation-related visual cues.

First, ROI occlusion-based sensitivity analysis was performed. The ROI annotation region was masked on each image, and patient-level predictions were recalculated. Performance on the original images was then compared with performance on ROI-occluded images to quantify the model’s sensitivity to visual information in and around the ROI region.

Second, a mask-only negative control analysis was conducted to test whether the ROI mask alone contained discriminative class information. In this analysis, anatomical image content was removed and classification was performed using only mask-derived information. Third, ROI geometry-based control was performed using limited non-image variables related to ROI position, size, and geometry. This analysis tested whether ROI placement itself carried systematic class-related information.

Finally, Grad-CAM-based visual explainability analysis was used to examine the regions contributing to model decisions. Grad-CAM heatmaps were visually assessed, and the overlap between the ROI annotation mask and Grad-CAM activation maps was quantified to estimate the extent to which model attention was concentrated within the annotation region.

Together, these analyses evaluated ROI-related effects through occlusion sensitivity, mask-only and geometry-only controls, and Grad-CAM attention analysis.

### 2.13. Post-Ensemble Clinical Scenario Analyses

In addition to the standard argmax-based classification results, two exploratory post-ensemble analyses were performed to evaluate how model outputs might behave in possible decision-support scenarios.

First, a threshold-based sensitivity analysis was performed for the Osteoporosis class. Different threshold values were applied to the Osteoporosis probability produced by the final ensemble model, and their effects on Osteoporosis recall, precision, macro-F1, and overall accuracy were evaluated. This analysis was intended to examine how the model would behave under a screening-oriented decision rule in which reducing false negatives may be prioritized.

Second, an abstention analysis based on model uncertainty was performed. The highest class probability and the margin between the two highest class probabilities were used as uncertainty measures. Patients below predefined confidence thresholds were considered abstained cases, and classification performance was recalculated for the remaining patient subgroup.

These analyses were exploratory and were not intended to define a clinical deployment threshold. They were used to characterize possible decision-support behavior, particularly for low-confidence or borderline cases that may require expert review.

### 2.14. Reporting Guidelines

This retrospective observational AI-based medical imaging study was reported with consideration of the STROBE recommendations for observational studies and the CLAIM 2024 checklist for artificial intelligence in medical imaging. These reporting guidelines were used to improve transparency in describing the study design, data source, reference standard, patient-level partitioning, model development, performance evaluation, artifact-control analyses, and study limitations. The completed CLAIM checklist is provided as Supplementary Table S1.

## 3. Results

Model performance was evaluated at the patient level using the predefined 5-fold stratified cross-validation structure. L1–L4 images from the same patient were kept together throughout all analyses. Therefore, the reported metrics are based on 190 patient-level predictions rather than 760 individual vertebral images. Performance was assessed using accuracy, balanced accuracy, macro-F1, weighted-F1, and class-wise precision, recall, and F1 scores. Because of class imbalance, macro-F1 and class-wise recall were interpreted together with overall accuracy.

### 3.1. Patient-Level Performance of Individual CNN Models

ResNet18, ResNet50, and DenseNet121 were first evaluated separately using the same patient-level L1–L4 feature-fusion structure. Among the individual models, ResNet18 achieved the highest overall performance, with an accuracy of 0.7158, macro-F1 of 0.7098, and weighted-F1 of 0.7163. ResNet50 showed slightly lower overall accuracy but produced the highest Osteoporosis F1 score among the individual models. DenseNet121 showed the lowest overall performance. The patient-level performance metrics of the individual CNN models are summarized in Table 5.

The individual model results indicate that three-class patient-level discrimination was particularly challenging between the Osteopenia and Osteoporosis classes. The relatively different class-wise behavior of ResNet18 and ResNet50 also suggested that the CNN backbones might provide partially complementary probability outputs. Therefore, ensemble strategies were further evaluated.

### 3.2. Comparison of Ensemble Strategies

Patient-level probability outputs from ResNet18, ResNet50, and DenseNet121 were combined using different ensemble strategies. The equal-weight soft-voting ensemble achieved the highest point-estimate performance, with an accuracy of 0.7474, macro-F1 of 0.7403, and weighted-F1 of 0.7472. This numerical advantage was interpreted cautiously because the paired comparisons did not show statistically definitive improvements over ResNet18 or ResNet50.

In paired comparisons, the improvements over ResNet18 and ResNet50 were not statistically definitive in this limited sample. For the final ensemble versus ResNet18, the exact McNemar test for accuracy difference yielded *p* = 0.3269, and the paired bootstrap 95% confidence interval for the macro-F1 difference ranged from −0.0214 to 0.0827. For the final ensemble versus ResNet50, the corresponding McNemar *p*-value was 0.2005, and the paired bootstrap 95% confidence interval for the macro-F1 difference ranged from −0.0198 to 0.0892. The difference compared with DenseNet121 was more pronounced, with an exact McNemar *p*-value of 0.0294 for accuracy difference and a paired bootstrap 95% confidence interval of 0.0112–0.1323 for the macro-F1 difference.

More complex combination strategies, including class-F1-weighted ensemble, class-wise calibrated grid, temperature-scaled equal ensemble, leakage-free logistic regression stacking, and ordinal isotonic post-processing, did not outperform the equal-weight soft-voting ensemble in terms of point estimates.

The results in Table 6 show that more complex meta-learner or calibration-based strategies did not provide a clear additional gain over simple equal-weight soft voting. The raw equal ensemble was therefore selected as the final model by considering its highest point-estimate performance, simpler structure, and interpretability under the patient-level evaluation protocol. However, this selection should not be interpreted as evidence of statistically confirmed superiority over all individual CNN backbones.

### 3.3. Performance of the Final Ensemble Model

The final equal-weight soft-voting ensemble combined patient-level probability outputs from ResNet18, ResNet50, and DenseNet121. Within the predefined patient-level 5-fold cross-validation framework, the final equal-weight soft-voting ensemble yielded an accuracy of 0.7474, balanced accuracy of 0.7377, macro-F1 of 0.7403, and weighted-F1 of 0.7472. In class-wise analysis, F1 scores were 0.7820 for Normal, 0.7485 for Osteopenia, and 0.6905 for Osteoporosis. For the Osteoporosis class, recall was 0.6744 and specificity was 0.9184. The class-wise performance metrics of the final ensemble model are presented in Table 7.

**Figure 7:**
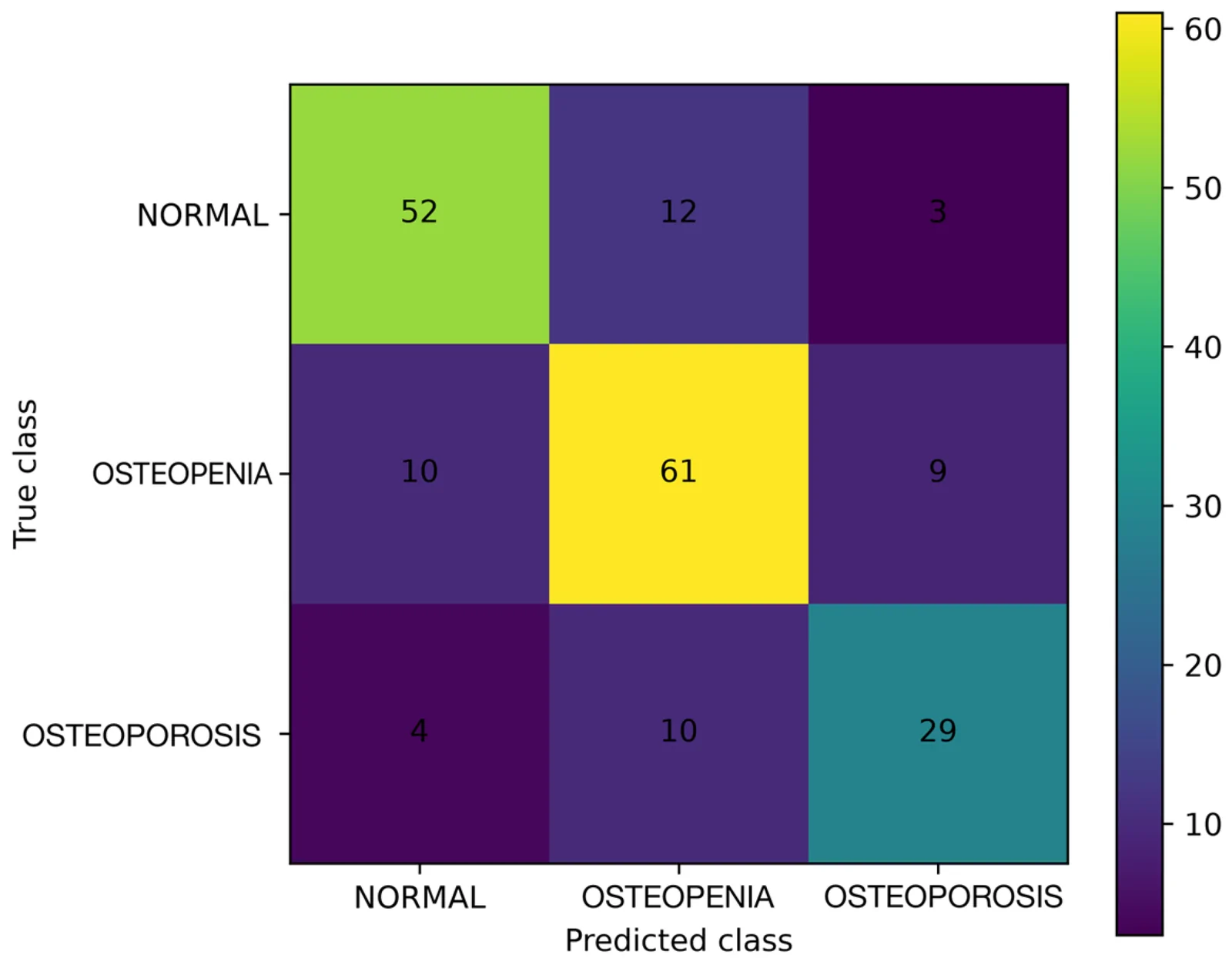
Patient-level confusion matrix of the final equal-weight soft-voting ensemble model. The model was evaluated on 190 patients. Rows indicate true classes and columns indicate predicted classes.

The confusion matrix of the final model is shown in Figure 7. The model correctly classified 52 of 67 Normal patients, 61 of 80 Osteopenia patients, and 29 of 43 Osteoporosis patients. The most frequent errors occurred between Osteopenia and Osteoporosis. Among Osteoporosis patients, 10 were classified as Osteopenia and 4 as Normal.

**Figure 8:**
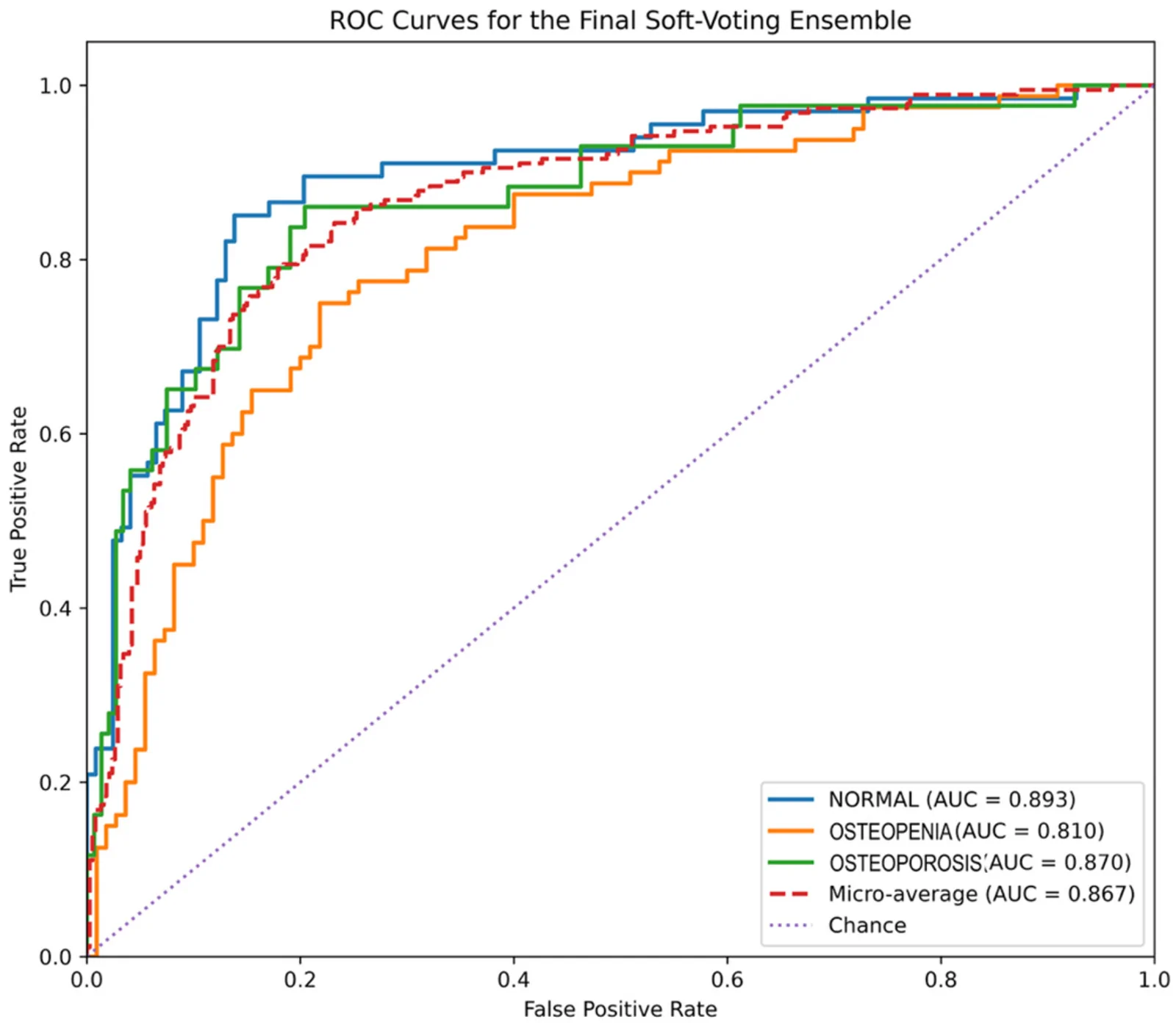
One-vs-rest ROC curves of the final equal-weight soft-voting ensemble model. ROC curves were generated separately for the Normal, Osteopenia, and Osteoporosis classes using patient-level prediction probabilities. The dashed curve denotes the micro-average ROC curve, and the dotted diagonal line indicates chance-level classification.

The class probabilities of the final model were further evaluated using one-vs-rest ROC-AUC analysis. AUC values were 0.8931 for Normal, 0.8099 for Osteopenia, and 0.8698 for Osteoporosis. Patient-level bootstrap 95% confidence intervals were 0.8382–0.9394 for Normal, 0.7441–0.8703 for Osteopenia, and 0.8011–0.9313 for Osteoporosis. The macro-average AUC was 0.8576 (95% CI: 0.8091–0.9013), and the weighted-average AUC was 0.8528 (95% CI: 0.8035–0.8964). These findings indicate that the final model captured a meaningful probability-level discriminative signal, with relatively lower discrimination for the intermediate Osteopenia class. The class-wise and average ROC-AUC values are summarized in Table 8, and the corresponding one-vs-rest ROC curves are shown in Figure 8.

### 3.4. Results of Artifact-Control and Visual Explainability Analyses

Because ROI annotations were retained on the images, additional artifact-control analyses were performed on the final equal-weight soft-voting ensemble. The aim was to evaluate whether model performance could be explained solely by ROI annotation or ROI geometry.

In the ROI occlusion analysis, the final model achieved an accuracy of 0.7474 and macro-F1 of 0.7403 on the original images. When the ROI region was occluded, accuracy decreased to 0.6737 and macro-F1 decreased to 0.6740. This corresponds to a decrease of 0.0737 in accuracy and 0.0663 in macro-F1. The performance change after ROI occlusion is summarized in Table 9.

After ROI occlusion, the final class prediction changed in 25 of 190 patients, corresponding to a prediction change rate of 0.1316. The accuracy decrease was statistically significant according to the exact McNemar test (*p* = 0.0066). The paired bootstrap 95% confidence interval for the macro-F1 difference was 0.0178–0.1146. The mean change in true-class probability was −0.0339, and the mean absolute change was 0.0914. These findings indicate that the ROI region and surrounding image content had a measurable effect on both final predictions and probability distributions.

Negative control analyses were then performed to test whether ROI annotation information alone was sufficient for class discrimination. Control models based only on the ROI mask achieved an accuracy of 0.3526 and macro-F1 of 0.1738. The ROI geometry-based control produced the same accuracy and macro-F1 values. These results indicate that neither the ROI mask nor ROI geometry alone provided meaningful discriminative information for distinguishing Normal, Osteopenia, and Osteoporosis classes. The mask-only and ROI geometry-only negative control results are presented in Table 10.

Grad-CAM analysis was used to examine the spatial distribution of model attention. For the ensemble mean, the annotation attention ratio was 0.0234, whereas the annotation area ratio was 0.0124. The annotation attention enrichment value was 1.8813, indicating that attention within the annotation region was higher than expected based on its relative area. However, the total attention located within the annotation region remained low, at approximately 2.34%. The Grad-CAM annotation overlap metrics are summarized in Table 11.

**Figure 9:**
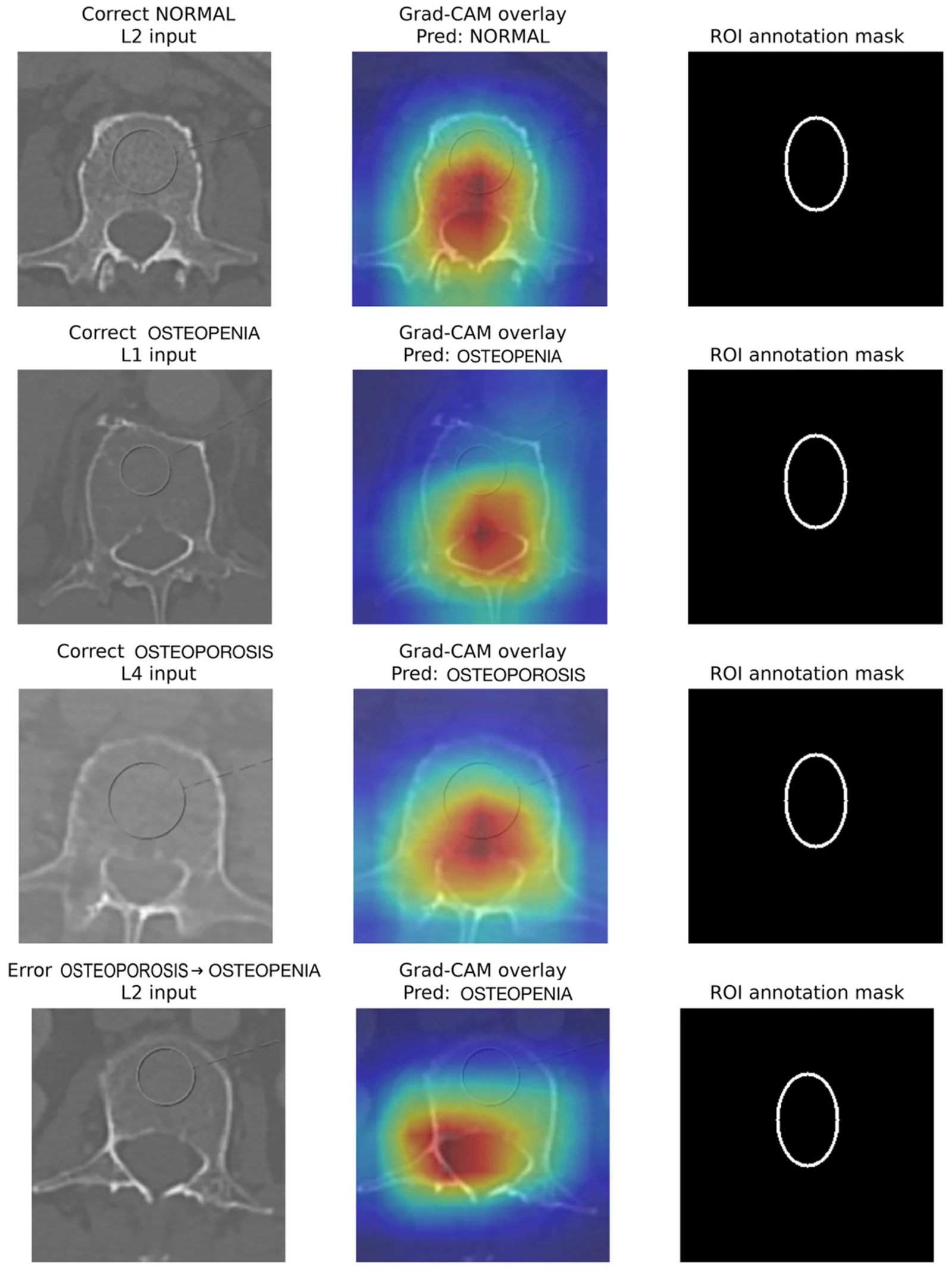
Grad-CAM-based artifact-control examples from the final ensemble model. Each row shows one patient example with the model input, Grad-CAM overlay, and ROI annotation mask. The circular/elliptical ring indicates the manually placed ROI annotation used during dataset preparation. The examples show that model activations were not confined to the ROI annotation but were distributed across the vertebral body and adjacent trabecular/cortical regions.

In class-wise Grad-CAM evaluation, annotation attention ratios for the ensemble mean were 0.0250 for Normal, 0.0218 for Osteopenia, and 0.0239 for Osteoporosis, indicating that ROI-related attention overlap was not strongly concentrated in a single class. Representative examples are shown in Figure 9. In correctly classified cases, activations were not confined to the ROI ring and were distributed across the vertebral body, including trabecular regions and cortical margins. In the Osteoporosis-to-Osteopenia misclassification example, the activation pattern was more diffuse and less class-discriminative.

Together, the artifact-control analyses indicate that final model performance cannot be explained solely by ROI annotation or ROI geometry. The model showed measurable sensitivity to the ROI region, but the negative controls and Grad-CAM results support that vertebral image content also contributed to the predictions.

### 3.5. Clinical Scenario Analyses

In addition to the standard argmax-based classification result, two exploratory clinical scenario analyses were performed: an Osteoporosis-sensitive threshold analysis and an abstention analysis based on prediction uncertainty.

In the Osteoporosis threshold analysis, the standard argmax approach produced an Osteoporosis recall of 0.6744. This corresponded to 14 false-negative Osteoporosis cases among 43 Osteoporosis patients. Lowering the Osteoporosis probability threshold increased recall. At the 0.26 threshold, Osteoporosis recall increased to 0.7674, while accuracy decreased to 0.7211 and macro-F1 decreased to 0.7193. At this threshold, the number of false negatives for Osteoporosis decreased from 14 to 10, while false positives increased from 12 to 21. Selected results from the Osteoporosis-sensitive threshold analysis are shown in Table 12.

In the abstention analysis, low-confidence cases were separated from the automatic classification pathway using either the highest class probability or the Top1–Top2 probability margin. When a maximum-probability threshold of 0.64 was used, patient coverage decreased to 0.7211; 137 patients remained in the automatic classification set and 53 were assigned to the abstention group. In the classified subgroup, accuracy increased to 0.7956, macro-F1 to 0.7913, and Osteoporosis F1 to 0.7541.

A similar pattern was observed with the Top1–Top2 margin criterion. At a margin threshold of 0.32, coverage was 0.7263, with 138 patients classified automatically and 52 assigned to abstention. In this subgroup, accuracy was 0.7899, macro-F1 was 0.7862, and Osteoporosis F1 was 0.7541. Selected abstention-based decision-support scenarios are summarized in Table 13.

Overall, the clinical scenario analyses suggest that model outputs may be more appropriately interpreted within a screening and prioritization framework, particularly when probability thresholds and uncertainty-aware abstention rules are considered alongside the main argmax-based result.

## 4. Discussion

### 4.1. Principal Findings

This study evaluated whether Normal, Osteopenia, and Osteoporosis classes could be distinguished at the patient level using L1–L4 lumbar vertebral PNG images derived from routine thoracoabdominal CT examinations. The study addressed a practical but challenging data scenario in which raw DICOM data, true HU values, and quantitative BMD information were not available for model development. Within this setting, patient-level multi-vertebral transfer learning provided moderate but meaningful discriminative performance.

Within the predefined patient-level 5-fold cross-validation framework, the final equal-weight soft-voting ensemble yielded an accuracy of 0.7474, balanced accuracy of 0.7377, macro-F1 of 0.7403, weighted-F1 of 0.7472, and macro-average ROC-AUC of 0.8576. These findings indicate that CT-derived PNG images retain image-level signals relevant to three-class bone density category discrimination, even when quantitative densitometric information is unavailable. The performance level should not be interpreted as evidence for direct diagnostic deployment; rather, it supports the feasibility of an image-based decision-support approach in a constrained but clinically plausible data format, consistent with recent reviews emphasizing both the promise of AI-based osteoporosis screening and the need for cautious validation [9,10,11].

A key strength of the study is that the evaluation was conducted at the patient level rather than at the individual image level. Each patient was represented by L1, L2, L3, and L4 images, and these four images were kept together throughout training and evaluation. This design prevented patient-level data leakage and allowed model outputs to be interpreted in a manner closer to the clinical decision unit. In addition, the study did not rely only on point-estimate classification metrics. Bootstrap confidence intervals, paired statistical comparisons, ROC-AUC analysis, threshold-based sensitivity analysis, abstention analysis, and artifact-control experiments were included to provide a more transparent interpretation of model behavior.

### 4.2. Patient-Level Multi-Vertebral Modeling and Ensemble Behavior

The use of patient-level multi-vertebral representation was central to the design of this study. Instead of treating L1–L4 images as independent samples, the four vertebral levels from each patient were processed together and fused into a single patient-level representation. This structure was important for two reasons. First, it avoided the overestimation of performance that can arise when different images from the same patient are distributed across training and validation folds. Second, it allowed the model to use complementary anatomical and textural information from multiple lumbar levels rather than depending on a single vertebral slice.

The multi-vertebral design should be interpreted as a methodological framework rather than as proof that L1–L4 fusion is superior to every single-vertebral approach. Single-level ablation experiments were not performed in this study, and therefore the independent contribution of each vertebral level remains to be investigated. Nevertheless, the chosen design is well aligned with the patient-level organization of the dataset and with the aim of reducing image-level leakage in a limited medical imaging cohort.

Among the individual CNN backbones, ResNet18 provided the most balanced overall performance, whereas ResNet50 showed relatively stronger performance for the Osteoporosis class. This pattern suggests that different CNN backbones may capture partially different aspects of the vertebral image signal. The equal-weight soft-voting ensemble provided the highest point-estimate performance among the tested models and combination strategies. However, paired comparisons showed that the improvements over ResNet18 and ResNet50 were not statistically definitive in this limited sample. Therefore, the ensemble should be interpreted as a transparent and numerically favorable aggregation strategy rather than as a model with statistically confirmed superiority over the stronger individual backbones. The difference compared with DenseNet121 was more pronounced, but the overall interpretation should remain cautious.

More complex combination strategies, including class-based weighting, temperature scaling, leakage-free logistic regression stacking, and ordinal isotonic post-processing, did not outperform the equal-weight soft-voting ensemble. This finding is methodologically relevant. In small or moderate-sized medical imaging datasets, more flexible meta-learners may not necessarily improve generalization, even when they appear attractive from a modeling perspective. In the present study, the simpler ensemble strategy offered the best balance between performance, transparency, and interpretability for the patient-level cross-validation framework used in this study.

The ROC-AUC analysis further supports this interpretation. The final ensemble showed stronger probability-level discrimination for the Normal and Osteoporosis classes than for Osteopenia. This is consistent with the clinical nature of osteopenia as an intermediate category between normal bone density and osteoporosis. The lower AUC and the confusion matrix patterns observed for Osteopenia therefore appear clinically plausible rather than unexpected. This is also consistent with previous three-class CT-based osteoporosis studies, in which distinguishing osteopenia from adjacent bone-density categories has required more complex modeling strategies, including feature fusion, localization, segmentation, or multimodal inputs [14,15,16].

### 4.3. Artifact-Control Analyses and Model Reliability

The presence of visible ROI annotations on the PNG images was an important methodological issue in this study. These annotations were created during radiological assessment and were not provided to the model as masks, additional channels, or explicit classification features. They were used only as anatomical references for determining the standardized crop center. However, because the ROI lines remained visible after cropping, their possible influence on model decisions had to be evaluated.

Rather than ignoring this issue, the study treated ROI annotations as a potential source of bias and tested their influence through complementary artifact-control analyses. The ROI occlusion analysis showed a measurable performance decrease when the ROI region was masked. This finding indicates that the ROI region was not neutral for the model and that visual information in or around this region influenced patient-level predictions. Therefore, residual ROI-related sensitivity should be considered an important limitation of the present dataset. However, the occlusion result should not be interpreted as evidence that the model relied only on the visible annotation line. The occluded region also included central vertebral trabecular content and adjacent local image information, which are clinically relevant for bone-density assessment. Thus, ROI occlusion removes both potential annotation-related cues and real anatomical image information.

The negative control analyses were used to distinguish ROI-region sensitivity from a shortcut based solely on annotation shape or placement. Models trained only on the ROI mask or ROI geometry remained close to majority-class-level performance and did not provide meaningful class discrimination. This indicates that the final model performance cannot be explained solely by the visible ROI annotation or by its geometric placement. In other words, the ROI annotation or its geometry alone did not contain sufficient information to reproduce the observed patient-level classification performance.

Grad-CAM analysis provided a complementary visual interpretation. Model activations were not confined to the ROI ring; they were distributed across the vertebral body, including trabecular regions and cortical margins. At the same time, attention enrichment within the annotation region was greater than 1, indicating that the ROI area was not completely neutral from the model’s perspective. Taken together, these findings support a balanced interpretation: the model did not rely solely on ROI annotations or ROI geometry, but residual ROI-related sensitivity cannot be fully excluded.

Overall, the artifact-control framework strengthens the transparency of the study by making a potential source of bias measurable and discussable rather than leaving it unexamined. The results suggest that the final model used vertebral image content, but they also confirm that ROI-related visual factors may have influenced the predictions. Therefore, the retained ROI annotation should be interpreted as a controlled limitation, not as a fully eliminated source of bias. Future validation on raw DICOM-derived or annotation-free images would be the most direct way to reduce this uncertainty.

### 4.4. Clinical Interpretation and Decision-Support Scenarios

The most prominent error pattern in the final model occurred between the Osteopenia and Osteoporosis classes. This is clinically understandable because these categories represent adjacent regions along the bone density spectrum. Compared with the distinction between clearly normal and clearly osteoporotic cases, the visual separation of osteopenia from osteoporosis is expected to be more challenging, particularly when the input consists of CT-derived PNG images rather than quantitative HU or BMD measurements. This interpretation is compatible with CT-based quantitative and AI-assisted studies showing that density-related information is more directly captured when HU, QCT, or BMD-like measurements are available [7,12,13].

When raw DICOM data and reliable HU measurements are available, simple HU-based assessment, QCT, or DL-BMD approaches may provide more direct, quantitative, and clinically interpretable information about bone density than a PNG-based visual classification model. Therefore, the proposed model should not be considered an alternative to HU measurement, QCT, or BMD estimation in settings where these quantitative data are available. Its practical relevance lies in a narrower scenario: anonymized or retrospectively exported CT-derived PNG images in which DICOM metadata, true HU values, scanner calibration information, and quantitative density measurements are not preserved. In this setting, the model evaluates whether patient-level visual patterns from L1–L4 vertebral images still contain discriminative information for Normal, Osteopenia, and Osteoporosis classification.

The use of the L1–L4 composite/mean DXA T-score as the reference label was chosen to match the patient-level multi-vertebral image representation used in the model. Because the model jointly analyzed L1, L2, L3, and L4 images, a lumbar spine-level DXA reference was considered more anatomically consistent than assigning labels from a single vertebral level. However, lumbar DXA may be affected by degenerative changes, osteophytes, vertebral deformity, or fracture. For this reason, patients with advanced degenerative changes and/or prominent osteophytes, vertebral fracture, severe scoliosis or vertebral deformity, metallic implant/bone cement, vertebral metastasis or tumoral infiltration, infectious involvement, or inadequate image quality were excluded during patient selection.

This error pattern is important for defining the appropriate clinical role of the model. The results do not support using the model as a stand-alone diagnostic system that produces definitive decisions for all patients. Instead, the findings are more consistent with a screening- and prioritization-oriented decision-support role. In such a scenario, model outputs would be interpreted together with confidence levels, and uncertain or borderline cases would be directed to expert review rather than being treated as automatic final classifications.

The Osteoporosis recall of 0.6744 under the standard argmax decision rule is clinically important. In practical terms, 14 of 43 Osteoporosis patients were classified into either Normal or Osteopenia classes. For a screening-oriented model, this false-negative rate is a major limitation because missed Osteoporosis cases may not be referred for further DXA assessment, clinical evaluation, or treatment consideration. Therefore, the current model should not be interpreted as a stand-alone screening tool capable of safely excluding Osteoporosis.

The threshold-based analysis for the Osteoporosis class illustrates how this limitation could be managed in a decision-support setting. Lowering the Osteoporosis probability threshold increased Osteoporosis recall from 0.6744 to 0.7674 and reduced Osteoporosis false negatives from 14 to 10. However, this gain was accompanied by an increase in false-positive Osteoporosis predictions and a decrease in overall accuracy and macro-F1. Therefore, threshold selection should depend on the intended clinical workflow and on the acceptable balance between false negatives and false positives. In its current form, the model is more appropriately positioned as a prioritization aid that may flag patients for expert review rather than as an autonomous rule-out tool for Osteoporosis.

The abstention analysis provided another decision-support perspective. When low-confidence cases were separated from the automatic classification pathway, performance improved in the remaining patient subgroup. This suggests that the model may be more useful when paired with an uncertainty-aware workflow. Rather than forcing a decision for every patient, the model could support prioritization by identifying cases where automatic classification is more reliable and cases where expert evaluation is more appropriate.

These clinical scenario analyses should be interpreted as exploratory. They do not establish a clinical deployment threshold or validate a complete clinical workflow. However, they help define how a moderate-performance model can be positioned more safely and realistically: not as a replacement for established diagnostic assessment, but as a possible image-based decision-support component requiring further validation.

### 4.5. Limitations and Future Directions

Several limitations should be considered when interpreting the findings. First, the study used CT-derived PNG images rather than raw DICOM data. As a result, true HU values, windowing parameters, scanner metadata, and quantitative density information were not available for modeling. Therefore, the proposed approach cannot be interpreted as HU-based measurement, QCT-like BMD estimation, or BMD regression. When DICOM data and reliable HU measurements are available, HU-based or QCT/DL-BMD-based approaches may be more appropriate for quantitative osteoporosis assessment. The present study specifically evaluates a narrower image-based classification scenario in which only exported anonymized vertebral images are available. This limitation also defines the practical relevance of the work, because such non-DICOM image formats may be encountered in retrospective image archives or restricted data-sharing settings where DICOM-based quantitative measurements cannot be reconstructed.

Second, best-epoch selection and final performance reporting were not fully separated within the cross-validation design. In each fold, the best training epoch/checkpoint for each model was selected according to validation macro-F1, and the resulting predictions from that same fold were subsequently used for fold-level performance reporting. Although patient-level data were never shared between the training and validation partitions of a given fold, and therefore no inter-partition patient or image overlap occurred, the checkpoint-selection criterion and the reported performance estimates were derived from the same fold. Consequently, these quantities are not statistically independent, which may introduce a degree of optimistic bias into the reported performance estimates. A more conservative evaluation design would separate model selection from final evaluation, for example by reserving an internal validation subset within the training portion for checkpoint selection while using the outer held-out fold exclusively for performance evaluation, or by adopting nested cross-validation. Such a design was not implemented in the present study. Therefore, the reported values should be interpreted as cross-validation performance estimates rather than as results from a strictly independent held-out test set. Future studies should use nested cross-validation, a dedicated internal validation subset, and ultimately independent external validation to obtain more conservative and generalizable performance estimates.

Third, the dataset was obtained from a single center and included 190 patients, with fewer Osteoporosis cases than Normal and Osteopenia cases. This sample size is limited for the development and evaluation of multiple deep learning models, particularly for a three-class medical imaging problem in which the smallest class contained 43 patients. To reduce the risk of overfitting under these conditions, the study used ImageNet-pretrained CNN backbones, conservative data augmentation, class-weighted loss, patient-level cross-validation, macro-F1-based model selection, and bootstrap confidence intervals. Nevertheless, these measures cannot fully compensate for the limited sample size or establish broad generalizability. In addition, the study did not include an independent external validation cohort. Although patient-level cross-validation was used to estimate model performance while reducing data leakage, cross-validation remains an internal validation strategy and cannot fully demonstrate generalizability to new institutions, scanners, acquisition protocols, or patient populations. Therefore, the reported performance should be interpreted as internal preclinical feasibility evidence rather than as externally validated clinical performance.

Fourth, all CT images were acquired using the same scanner and the same institutional imaging protocol. This design reduced technical heterogeneity within the dataset and made the internal analysis more controlled, but it also limited the ability to evaluate scanner- and protocol-related robustness. CT image appearance may vary across scanner manufacturers, reconstruction kernels, slice thicknesses, dose settings, image export procedures, and windowing conditions. Because the present study used exported PNG images rather than raw DICOM data, such acquisition- and export-related differences may have an even greater effect on model behavior. Therefore, the current results cannot be assumed to transfer directly to images acquired with different scanners or protocols. External validation using multicenter, multi-scanner, and protocol-diverse datasets is required before clinical implementation.

Fifth, the moderate recall for the Osteoporosis class under the standard argmax decision rule is a clinically important limitation. Because a screening-oriented model should minimize missed Osteoporosis cases, the observed false-negative rate limits the direct clinical applicability of the model. Future studies should optimize and validate screening-oriented decision thresholds in independent cohorts, with explicit consideration of the trade-off between false negatives and false positives.

Sixth, ROI annotations were visible on the input images and all ROI placements were performed by a single experienced radiologist. Although the ROI annotations were not used as direct model inputs and artifact-control analyses were performed, the ROI occlusion experiment demonstrated that the ROI region had a measurable influence on model predictions. Therefore, ROI-related visual sensitivity cannot be completely removed in the present dataset and should be considered a limitation. The mask-only and geometry-only controls suggested that annotation shape or placement alone was insufficient to explain the model performance, but these controls do not fully eliminate the possibility of residual ROI-related effects. Inter-reader variability in ROI placement was also not assessed. Future work should evaluate the same patient-level multi-vertebral architecture on DICOM-based or annotation-free images and, where applicable, include multi-reader annotation protocols.

Finally, although age, sex, and CT–DXA interval were reported to describe the study cohort, the model was developed using image data only. Age, sex, clinical risk factors, laboratory findings, treatment history, numerical DXA/T-score values, and CT–DXA interval were not included as model inputs. In routine clinical practice, osteoporosis and osteopenia assessment is not based solely on image appearance, but on a broader clinical context. Future studies should therefore investigate multimodal models that integrate imaging features with clinical variables, while also comparing image-based predictions with HU, QCT, and BMD-derived measures, as suggested by recent multimodal and CT-based osteoporosis screening studies [8,18,21]. Such studies would help determine whether patient-level multi-vertebral image representation can contribute additional information to established osteoporosis screening workflows.

Overall, the present study provides controlled preclinical feasibility evidence for patient-level three-class osteoporosis screening from CT-derived PNG images. Its value lies not in replacing quantitative densitometry, but in demonstrating that a patient-level, multi-vertebral, artifact-controlled AI workflow designed to prevent inter-partition patient overlap can extract relevant image-based signals from a constrained clinical image format. Further validation on larger, DICOM-based, annotation-free, and multicenter datasets is needed before translation into clinical decision-support practice.

## 5. Conclusions

In this study, we presented a deep learning approach based on multi-vertebral representation to evaluate patient-level discriminability of the Normal, Osteopenia, and Osteoporosis classes using thoracoabdominal CT-derived lumbar vertebral PNG images. Each patient was represented by the L1–L4 vertebral levels, and a patient-level cross-validation protocol preventing data leakage was applied by ensuring that images from the same patient were not separated during training and evaluation.

The findings showed that combining patient-level probabilities obtained from individual CNN models using an equal-weight soft-voting ensemble yielded the highest point-estimate performance among the tested strategies. However, this numerical advantage should be interpreted cautiously because the improvement over ResNet18 and ResNet50 was not statistically definitive. Under the patient-level 5-fold cross-validation protocol, the final ensemble model reached a cross-validation accuracy of 0.7474, macro-F1 of 0.7403, and weighted-F1 of 0.7472, while the one-vs-rest ROC-AUC analysis yielded a macro-average AUC of 0.858; as discussed in Section 4.5, these values should be interpreted as cross-validation performance estimates rather than as results from an independent, held-out test set. This performance indicates that a meaningful image-based signal for patient-level three-class bone density category discrimination can be obtained from CT-derived PNG images without using HU or BMD information.

The potential artifact effect of ROI annotations was also evaluated in this study. The ROI occlusion analysis showed that the model was influenced by local image information in and around the ROI region. However, the mask-only and geometry-only negative controls demonstrated that model performance could not be explained solely by ROI annotation or ROI geometry. Grad-CAM analyses also supported that model activations were not confined to the ROI ring, but were distributed across the vertebral body and related anatomical regions. Nevertheless, it was acknowledged that residual ROI-related effects could not be fully excluded, and this was considered one of the important limitations of the study.

The clinical scenario analyses indicated that the model may be more appropriately positioned as a decision-support component interpreted together with confidence levels, rather than as a system that produces automatic and definitive decisions for all patients. The osteoporosis-sensitive threshold analysis showed that the decision threshold could be adjusted according to clinical priorities in screening scenarios aimed at reducing false negatives. The abstention analysis further showed that when low-confidence patients were directed to expert review, classification performance increased in the remaining patient subgroup.

In conclusion, this study demonstrates that patient-level multi-vertebral representation from thoracoabdominal CT-derived lumbar vertebral PNG images has potential for osteoporosis screening and decision-support research, even in the absence of raw DICOM data and quantitative HU or BMD information. The findings should therefore be interpreted as preclinical feasibility evidence obtained under a patient-level cross-validation design in which the same fold was used for model selection and performance reporting, rather than as externally validated clinical performance. Independent external validation in multicenter patient populations, together with integration of relevant clinical variables, is required before clinical implementation. In its current form, the study supports the investigational value of leakage-free, patient-level, multi-vertebral, image-based artificial intelligence approaches for distinguishing Normal, Osteopenia, and Osteoporosis classes.

## Figures and Tables

**Table 1:** Patient characteristics, class distribution, and CT–DXA interval.

Group	Number of Patients	Number of Images	Age, Years, Mean ± SD	Female, n (%)	Male, n (%)	CT–DXA Interval, Days, Mean ± SD
Normal	67	268	66.5 ± 10.6	49 (73.13%)	18 (26.87%)	39.8 ± 26.1
Osteopenia	80	320	65.1 ± 10.8	71 (88.75%)	9 (11.25%)	44.2 ± 29.7
Osteoporosis	43	172	66.8 ± 8.7	39 (90.70%)	4 (9.30%)	43.9 ± 29.1
Total	190	760	-	159 (83.68%)	31 (16.32%)	42.6 ± 28.4

**Table 2:** Patient-level 5-fold stratified cross-validation distribution.

Fold	Normal	Osteopenia	Osteoporosis	Total
0	14	16	8	38
1	14	16	8	38
2	13	16	9	38
3	13	16	9	38
4	13	16	9	38

**Table 3:** CNN backbones and patient-level feature-fusion structures.

Model	Pretraining	Feature Dimension per Vertebra	Patient-Level Concatenated Feature Dimension	Classifier
ResNet18	ImageNet	512	2048	FC → ReLU → Dropout → FC → ReLU → Dropout → FC
ResNet50	ImageNet	2048	8192	FC → ReLU → Dropout → FC → ReLU → Dropout → FC
DenseNet121	ImageNet	1024	4096	FC → ReLU → Dropout → FC → ReLU → Dropout → FC

**Table 4:** Training hyperparameters used for the CNN models.

Parameter	ResNet18	ResNet50	DenseNet121
Input size	224 × 224	224 × 224	224 × 224
Batch size	8	8	8
Optimizer	AdamW	AdamW	AdamW
Learning rate	1 × 10^−4^	5 × 10^−5^	1 × 10^−4^
Weight decay	1 × 10^−4^	1 × 10^−4^	1 × 10^−4^
Dropout	0.40	0.50	0.40
Maximum epochs	50	60	40
Early stopping patience	12	15	12
Scheduler	ReduceLROnPlateau	ReduceLROnPlateau	ReduceLROnPlateau
Model selection	Val. macro-F1	Val. macro-F1	Val. macro-F1
Loss function	Weighted CE	Weighted CE	Weighted CE

**Table 5:** Patient-level performance of individual CNN models.

Model	Accuracy	Balanced Accuracy	Macro-F1	Weighted-F1	Normal F1	Osteopenia F1	Osteoporosis F1	Osteoporosis Recall
ResNet18	0.7158	0.7067	0.7098	0.7163	0.7597	0.7108	0.6588	0.6512
ResNet50	0.7053	0.7019	0.7050	0.7052	0.7206	0.6957	0.6988	0.6744
DenseNet121	0.6789	0.6671	0.6703	0.6791	0.7087	0.6905	0.6118	0.6047

**Table 6:** Comparison of ensemble and meta-learner strategies.

Method	Accuracy	Balanced Accuracy	Macro-F1	Weighted-F1	Osteoporosis F1	Osteoporosis Recall
Equal-weight soft-voting ensemble	0.7474	0.7377	0.7403	0.7472	0.6905	0.6744
Class-F1 weighted ensemble	0.7368	0.7277	0.7311	0.7368	0.6905	0.6744
Logistic stacking	0.7263	0.7111	0.7159	0.7248	0.6500	0.6047
Class-wise calibrated grid	0.7263	0.7103	0.7156	0.7254	0.6420	0.6047
Temperature-scaled equal ensemble	0.7211	0.7108	0.7145	0.7211	0.6667	0.6512
Ordinal isotonic post-processing	0.7000	0.6723	0.6811	0.6953	0.5753	0.4884

**Table 7:** Class-wise performance of the final equal-weight soft-voting ensemble model.

Class	Precision	Recall	F1	Specificity
Normal	0.7879	0.7761	0.7820	0.8862
Osteopenia	0.7349	0.7625	0.7485	0.8000
Osteoporosis	0.7073	0.6744	0.6905	0.9184
Macro-average	0.7434	0.7377	0.7403	0.8682

**Table 8:** Class-wise and average ROC-AUC values of the final equal-weight soft-voting ensemble model. Values in parentheses indicate patient-level bootstrap 95% confidence intervals.

Class/Average	ROC-AUC
Normal	0.8931 (0.8382–0.9394)
Osteopenia	0.8099 (0.7441–0.8703)
Osteoporosis	0.8698 (0.8011–0.9313)
Macro-average	0.8576 (0.8091–0.9013)
Weighted-average	0.8528 (0.8035–0.8964)
Micro-average	0.8666 (0.8219–0.9061)

**Table 9:** Change in final model performance in the ROI occlusion analysis.

Condition	Accuracy	Balanced Accuracy	Macro-F1	Weighted-F1	Normal F1	Osteopenia F1	Osteoporosis F1	Osteoporosis Recall
Original images	0.7474	0.7377	0.7403	0.7472	0.7820	0.7485	0.6905	0.6744
ROI-occluded images	0.6737	0.6896	0.6740	0.6743	0.7287	0.6400	0.6535	0.7674
Change	−0.0737	−0.0480	−0.0663	−0.0728	−0.0533	−0.1085	−0.0370	+0.0930

**Table 10:** Negative control analyses based on ROI mask and ROI geometry.

Control Analysis	Classifier	Accuracy	Balanced Accuracy	Macro-F1	Weighted-F1
Mask-only	PCA + Logistic regression	0.3526	0.3333	0.1738	0.1839
Mask-only	Random forest	0.3526	0.3333	0.1738	0.1839
ROI geometry-only	Logistic regression	0.3526	0.3333	0.1738	0.1839
ROI geometry-only	Random forest	0.3526	0.3333	0.1738	0.1839

**Table 11:** Grad-CAM-based overlap between model attention and ROI annotation regions.

Model	Number of Records	Annotation Attention Ratio	Annotation Area Ratio	Attention Enrichment
ResNet18	760	0.0192	0.0124	1.5471
ResNet50	760	0.0374	0.0124	3.0071
DenseNet121	760	0.0187	0.0124	1.5037
Ensemble mean	760	0.0234	0.0124	1.8813

**Table 12:** Selected results from the osteoporosis-sensitive threshold analysis.

Osteoporosis Threshold	Accuracy	Macro-F1	Osteoporosis Precision	Osteoporosis Recall	Osteoporosis F1	False Negatives	False Positives
Argmax/final model	0.7474	0.7403	0.7073	0.6744	0.6905	14	12
0.26	0.7211	0.7193	0.6111	0.7674	0.6804	10	21
0.36	0.7263	0.7193	0.6444	0.6744	0.6591	14	16
0.48	0.7474	0.7388	0.7179	0.6512	0.6829	15	11

These findings show that lowering the Osteoporosis threshold can improve sensitivity in a screening-oriented scenario, but at the cost of more false-positive referrals and lower overall performance.

**Table 13:** Selected decision-support scenarios from the abstention analysis.

Abstention Rule	Threshold	Coverage	Classified Patients	Abstained Patients	Accuracy	Macro-F1	Osteoporosis F1
None/final model	-	1.0000	190	0	0.7474	0.7403	0.6905
Max probability	0.64	0.7211	137	53	0.7956	0.7913	0.7541
Max probability	0.62	0.7368	140	50	0.7857	0.7826	0.7500
Top1–Top2 margin	0.32	0.7263	138	52	0.7899	0.7862	0.7541
Top1–Top2 margin	0.30	0.7579	144	46	0.7847	0.7797	0.7385

## Data Availability

The data that support the findings of this study are not publicly available due to ethical and privacy restrictions related to patient imaging data. De-identified derived tables, including patient-level prediction outputs and statistical analysis results, may be made available from the corresponding author upon reasonable request, subject to institutional approval and the conditions of the ethics committee decision.

## Supplementary Materials

The following supporting information can be downloaded at: https://www.mdpi.com/article/10.3390/biomedicines14091988/s1, Table S1: Completed CLAIM 2024 Checklist.

## Abbreviations

The following abbreviations are used in this manuscript: AI, artificial intelligence; AUC, area under the curve; BMD, bone mineral density; CNN, convolutional neural network; CT, computed tomography; DICOM, Digital Imaging and Communications in Medicine; DXA, dual-energy X-ray absorptiometry; HU, Hounsfield unit; QCT, quantitative computed tomography; ROI, region of interest; ROC, receiver operating characteristic; ROC-AUC, area under the receiver operating characteristic curve.
