# Supplementary material for: Artifact-Controlled Multi-Vertebral Transfer Learning Ensemble for Patient-Level Three-Class Osteoporosis Screening Using Thoracoabdominal CT-Derived Lumbar Images
Source: Biomedicines. 2026 Sep 3;14(9):1988. doi: 10.3390/biomedicines14091988 (PMC13604115; doi:10.3390/biomedicines14091988)
Supplement: Supplementary file 1 [file biomedicines-14-01988-s001.zip › biomedicines-4512927-supplementary.pdf]

Supplementary Table S1. Completed CLAIM 2024 Checklist

Artifact-Controlled Multi-Vertebral Transfer Learning En-semble for Patient-Level Three-Class Osteoporosis Screening Using Thoracoabdominal CT-Derived Lumbar Images

Checklist used: Checklist for Artificial Intelligence in Medical Imaging (CLAIM) 2024 update. The item wording below is summarized for completion of the checklist. Page and line numbers should be finalized after journal formatting.

Status legend: Yes = reported; Partially = partly reported or requires additional detail; No = not reported/not performed; Not applicable = not applicable to this retrospective non-interventional study.

Ethics statement: The study was approved by the Sivas Cumhuriyet University Health Sciences Research Ethics Committee (decision no: 2026-07/60; date: 16 July 2026). According to the ethics committee decision file, the submitted research documents were reviewed and the study was approved as ethically and scientifically appropriate. The ethics application file lists a Turkish-language Informed Voluntary Consent Form among the submitted documents, and an English translation of this form is also provided as a supplementary document The decision file does not contain a separate statement explicitly waiving the requirement for informed consent.

CLAIM item	Section	Checklist item summary	Status	Manuscript location	Comment for this study
1	Title/Abstract	Identifies the work as an AI/deep learning medical imaging study.	Yes	Title; Abstract	The title and abstract identify transfer learning, deep learning, ensemble modeling, and CT-derived lumbar images.
2	Abstract	Summarizes design, data, methods, results, and conclusion.	Yes	Abstract; Data Availability Statement	The structured abstract reports the study background/objectives, dataset size, modality, patient-level splitting, models, performance metrics with confidence intervals, artifact-control analyses, and cautious conclusions.
3	Introduction	Provides clinical/scientific background and intended role of the AI approach.	Yes	Introduction	The introduction summarizes osteoporosis screening, DXA, CT opportunistic screening, AI literature, and the intended decision-support role.
4	Introduction	States study aims, objectives, and scientific question.	Yes	End of Introduction	The study aim is stated as patient-level discrimination of Normal, Osteopenia, and Osteoporosis from CT-derived PNG images.
5	Study Design	States whether the study is prospective or retrospective.	Yes	Section 2.1	The study is described as retrospective, observational, and non-interventional.
6	Study Design	Defines study goal and intended use of the classification model.	Yes	Abstract; Sections 1, 2.14, 4.6, 5	The work is positioned as three-class screening and decision-support feasibility, not direct diagnosis.
7	Data	Describes data sources and alignment with intended use.	Yes	Sections 2.1-2.2; Data Availability Statement	Data consist of anonymized L1-L4 PNG images derived from routine thoracoabdominal CT examinations; privacy restrictions are stated.
8	Data	Reports inclusion/exclusion criteria and patient selection context.	Partially	Section 2.1; Section 4.5	Adult patients from 2021-2024 with thoracoabdominal CT and DXA within 6 months are described. A detailed initial-screening flowchart and full exclusion counts are not yet provided.
9	Data	Describes preprocessing steps and image transformations.	Yes	Sections 2.3-2.6; Figures 3-4	Integrity checks, ROI detection, crop-center shift, 320x320 cropping, grayscale conversion, 224x224 resizing, and absence of enhancement/padding are reported.
10	Data	Describes selection of data subsets, crops, or regions used for modeling.	Yes	Sections 2.4-2.5; Figures 3-4	ROI-guided crop generation and the rationale for preserving vertebral context rather than isolating ROI interior are described.
11	Data	Describes de-identification and removal of protected information.	Partially	Section 2.1; Data Availability Statement	Anonymization and removal of identifying fields are stated. The study was approved by the Sivas Cumhuriyet University Health Sciences Research Ethics Committee (decision no: 2026-07/60; date: 16 July 2026). The exact institutional de-identification procedure is not described in technical detail.
12	Data	Describes handling of missing or unreadable data.	Yes	Section 2.3	Automated integrity checks confirmed complete L1-L4 images for all patients and no corrupted or unreadable files.
13	Data	Reports image acquisition protocol.	Yes	Section 2.2	Scanner manufacturer/model, detector type, kVp, Smart mA, gantry rotation time, reconstruction thickness, and MPR use are reported.
14	Reference Standard	Defines how the reference standard was obtained.	Yes	Section 2.1	Reference labels are based on DXA-derived mean L1-L4 T-score values and WHO criteria.
15	Reference Standard	Explains rationale for the reference standard.	Yes	Introduction; Section 2.1	DXA is introduced as the accepted reference method for BMD assessment and used for patient class labels.
16	Reference Standard	Reports source and expertise related to annotations/labels.	Partially	Sections 2.1, 2.5; Section 4.5	DXA-based labels are described. ROI annotations were placed by one experienced radiologist. Inter-reader consensus was not performed and is reported as a limitation.

CLAIM item	Section	Checklist item summary	Status	Manuscript location	Comment for this study
17	Reference Standard	Describes labeling/annotation of the testing data.	Yes	Sections 2.1, 2.3, 2.12	All folds use the same patient-level DXA reference labels; evaluation is performed against clinical reference classes.
18	Reference Standard	Reports inter- or intrarater variability where applicable.	No	Section 4.5	Inter-reader or intrarater reliability was not assessed; this is explicitly discussed as a limitation.
19	Data Partitions	Describes assignment to training/optimization/testing partitions.	Yes	Sections 2.3, 2.9; Table 2	Patient-level 5-fold stratified cross-validation with 38 patients per fold is described.
20	Data Partitions	States level at which partitions are disjoint.	Yes	Sections 2.3, 2.9	L1-L4 images from the same patient were never split across folds; partitions are disjoint at patient level.
21	Testing Data	Reports testing sample size and its determination.	Partially	Sections 2.1, 2.3; Table 2; Section 4.5	The final sample size and fold sizes are reported. No a priori sample-size or power calculation was performed.
22	Model	Provides detailed model description.	Yes	Sections 2.7-2.8; Table 3; Figure 5	CNN backbones, feature dimensions, patient-level feature fusion, classifier structure, and output classes are described.
23	Model	Reports software libraries, frameworks, packages, and hardware.	Partially	Section 2.10	PyTorch, torchvision, NumPy, pandas, scikit-learn, OpenCV, PIL, Matplotlib, OS, CPU, and GPUs are reported. Exact library version numbers are not provided.
24	Model	Describes parameter initialization.	Partially	Sections 2.8-2.9	ImageNet pretraining is reported. Initialization details for newly added classifier layers are not specified.
25	Training	Reports training procedure, augmentation, stopping criteria, objective, and hyperparameters.	Yes	Section 2.9; Table 4	Weighted cross-entropy, AdamW, learning rates, weight decay, dropout, epochs, early stopping, scheduler, augmentation, and model selection metric are reported.
26	Training	Explains final model selection method.	Yes	Sections 2.11, 3.2; Table 6	Final model selection is based on point-estimate performance, simplicity, interpretability, and leakage-free evaluation.
27	Training	Describes ensembling technique.	Yes	Section 2.11; Equation 1; Section 3.2	ResNet18, ResNet50, and DenseNet121 probabilities are combined using equal-weight soft voting.
28	Evaluation	Describes performance metrics and their relevance.	Yes	Section 2.12; Results	Accuracy, balanced accuracy, macro-F1, weighted-F1, class-wise precision/recall/F1, specificity, ROC-AUC, threshold, and abstention metrics are reported.
29	Evaluation	Reports statistical uncertainty and significance testing methods.	Yes	Section 2.12; Results Tables 5-13	Patient-level bootstrap confidence intervals, exact McNemar tests, and paired bootstrap comparisons are reported.
30	Evaluation	Includes robustness or sensitivity analyses.	Yes	Sections 2.13-2.14; Sections 3.4-3.5	ROI occlusion, mask-only/geometry-only controls, threshold analysis, abstention analysis, calibration/meta-learner checks, and Grad-CAM analyses are included.
31	Evaluation	Describes explainability or interpretability methods.	Yes	Section 2.13; Section 3.4; Figure 8; Table 11	Grad-CAM and annotation-attention overlap metrics are described and reported.
32	Evaluation	Reports evaluation on internal data.	Yes	Sections 2.3, 2.9, 3.1-3.5	Internal evaluation is performed using patient-level 5-fold stratified cross-validation.
33	Evaluation	Reports external testing or explains its absence.	Yes	Section 4.5	No external testing was performed; the lack of independent external validation is explicitly discussed as a limitation.
34	Evaluation	Clinical trial registration where applicable.	Not applicable	Section 2.1	The study is retrospective, observational, and non-interventional; no clinical trial registration applies.
35	Results - Data	Reports numbers of patients/examinations/images included and excluded.	Partially	Sections 2.1, 2.3; Table 1; Table 2	Final included numbers and class/fold distributions are reported. A complete flowchart of initially eligible and excluded cases is not included.
36	Results - Data	Reports demographic/clinical characteristics by partition/dataset.	No	Section 4.5	Age, sex, clinical risk factors, laboratory values, and other clinical variables were not used as model inputs and are not reported by partition.
37	Results - Model Performance	Reports final model performance with uncertainty.	Yes	Sections 3.1-3.5; Tables 5-13; Figures 6-8	Final and comparative performance metrics, class-wise results, ROC-AUC, confidence intervals, and scenario analyses are reported.
38	Results - Model Performance	Reports diagnostic/classification performance estimates and precision.	Yes	Tables 5-13; Figure 7	Classification metrics and ROC-AUC values with patient-level bootstrap 95% confidence intervals are reported.
39	Results - Model Performance	Provides failure analysis of incorrect results.	Yes	Section 3.3; Section 3.4; Figures 6 and 8	Confusion matrix, inter-class error patterns, misclassification example, and Grad-CAM-based interpretation are provided.
40	Discussion	Discusses limitations, bias, uncertainty, and generalizability.	Yes	Section 4.5	Limitations include PNG/non-DICOM format, small imbalanced dataset, no external testing, model-selection optimism, batch size, ROI artifacts, ImageNet pretraining, missing clinical variables, and DXA-CT interval.

CLAIM item	Section	Checklist item summary	Status	Manuscript location	Comment for this study
41	Discussion	Discusses implications for practice and intended clinical role.	Yes	Sections 4.4, 4.6, 5	The model is positioned as a decision-support and screening/prioritization component, not a stand-alone diagnostic tool.
42	Other Information	Provides study protocol or additional technical details where needed.	Partially	Methods; Supplementary Material	The manuscript provides extensive methodological details. A separate protocol or public technical repository is not currently specified.
43	Other Information	States availability of software, trained model, and/or data.	Partially	Data Availability Statement; Supplementary Material	Patient imaging data are not public due to ethical and privacy restrictions. De-identified derived tables may be available from the corresponding author upon reasonable request, subject to institutional approval and the conditions of the ethics committee decision. Public availability of code and trained models is not yet specified.
44	Other Information	Reports funding and support, including funder role.	Yes	Funding; Acknowledgements; Declarations	The manuscript states no external funding and acknowledges institutional GPU infrastructure support.

Note. This completed checklist reflects the current manuscript draft. Items marked Partially or No identify areas where additional reporting could further improve transparency, but they do not necessarily indicate a methodological flaw when the item is not applicable or the limitation is explicitly acknowledged.
